# Distance to central-place drives species-specific habitat selection in sympatric insectivorous birds

**DOI:** 10.1186/s40462-026-00676-4

**Published:** 2026-07-03

**Authors:** Wiebke Ullmann, Nadja Schedensack, Stephanie Kramer-Schadt, Marius Grabow, Conny Landgraf, Carolin Scholz, Jan Pufelski, Florian Jeltsch, Ran Nathan, Sivan Toledo, Martin Mayer

**Affiliations:** 1 Department of Plant Ecology and Nature Conservation, University of Potsdam, Neues Palaise 10, 14469 Potsdam, Germany; 2 Leibniz Institute for Zoo and Wildlife Research (IZW), Alfred-Kowalke-Str. 17, 10315 Berlin, Germany; 3 Institute of Ecology, Technische Universität Berlin, Rothenburgstrasse 12, 12165 Berlin, Germany; 4 Department of Ecology, Evolution and Behavior, Hebrew University of Jerusalem, Givat Ram, Jerusalem, 91904 Israel; 5 Blavatnik School of Computer Science, Tel Aviv University, Check Point Building, Tel Aviv, 69978 Israel; 6 Department of Forestry and Wildlife Management, University of Inland Norway, Koppang, Norway; 7 Department of Ecoscience, Aarhus University, Aarhus, Denmark

**Keywords:** Movement, Habitat selection, Agriculture, ATLAS tags, High-resolution tracking, Niche differentiation, Coexistence

## Abstract

**Background:**

Agricultural intensification is a major driver of land use change, thereby reducing biodiversity and leading to population declines across various animal groups. In response, animals can mediate some negative fitness impacts and navigate through agricultural landscapes with reduced resource availability by changing their habitat selection via movement decisions — particularly when constrained by central-place foraging, which requires balancing travel costs against energetic returns.

**Methods:**

Here, using miniature ATLAS tags (Advanced Tracking and Localisation of Animals in real-life Systems), we tracked 101 house martins and 87 barn swallows at high-resolution to investigate their state-specific habitat selection and mapped the insect abundance and diversity across an intensively used agricultural landscape.

**Results:**

Both species mainly avoided arable fields and increasingly selected for forests and water bodies with distance from the colonies. House martins ranged farther from colonies than barn swallows and also showed strong distance-dependent selection for structurally complex habitats, such as extensive grasslands and green areas within villages. Furthermore, house martins selected for proximity to water bodies, while barn swallows’ selection focused on proximity to woody vegetation structures. During our 2023 insect sampling window, habitat selection tracked mapped insect richness more closely than mapped insect abundance.

**Conclusions:**

Distance-dependent divergence in space use suggests horizontal niche differentiation between sympatric central-place foragers, with potential implications for coexistence. Combined, our findings point to the importance of maintaining extensively used grasslands and small-scale habitat structures within intensively managed farmland to improve the abundance and diversity of prey for farmland passerines.

**Supplementary Information:**

The online version contains supplementary material available at 10.1186/s40462-026-00676-4.

## Background

Agricultural land covers about half of the Earth’s habitable area [1], with Europe among the most intensively managed regions worldwide [2]. These landscapes are characterised by high pesticide and fertilizer inputs, large crop fields, and monocultures, creating homogeneous agricultural systems with few natural elements [3]. The resulting losses of habitat, structural complexity, and connectivity, and direct effects of agrochemicals, have led to declines in plant and animal diversity and abundance [4, 5], thereby impairing ecosystem functions and disrupting trophic networks [6]. In such simplified systems, prey availability for higher-level consumers declines not only in quantity [7, 8] but also in quality, which may strongly influence consumer fitness [9].

For higher-level consumers, adjusting movement and behaviour is key to coping with land-cover change and reduced resource availability. Such adjustments may include shifts in prey choice [10, 11], foraging patterns [12], movement responses to food availability [13] and habitat selection [14, 15], which are inherently reflected in movement behaviour [16]. In homogeneous agricultural landscapes, these behavioural adjustments often involve longer travel distances because profitable habitat patches are scarce or distant and food and shelter requirements are difficult to meet within the same area [17]. However, the energetic gain in distant foraging grounds should outweigh the cost of traveling, as predicted by central-place foraging theory: an extension of optimal foraging theory [18] in which patch value declines with distance from the central place as travel costs increase [19, 20]. How these central-place constraints play out when multiple sympatric species share a landscape is less well understood. Recent theoretical work by Rueffler and Lehmann [12] begins to address this, by proposing that species-specific differences in travel distance and habitat selection may themselves act as a behavioural coexistence mechanism. Their model suggests that by foraging at different distances or in different habitats around their central places, sympatric species generate distinct prey-depletion halos that spatially partition resource use and reduce interspecific competition, even where their resource requirements overlap. Empirical evidence consistent with this mechanism has mainly been shown for sympatric seabird assemblages [21]. For terrestrial central-place foragers, equivalent tests remain less common; recent GPS tracking in aerial insectivores has, however, begun to demonstrate distance-dependent habitat selection in studies of Tree Swallows [22] and Barn Swallows [14]. Harris et al., [14] compared sympatric Barn Swallows and Tree Swallows in agricultural landscapes and found broadly similar foraging strategies between the two species, with strong shared selection for distant wetland habitat. However, joint comparisons across sympatric species, behavioural states, and distance from the colony, the focus of the present study, remain scarce.

Typical terrestrial central-place foragers in industrialised agricultural landscapes are, for example, insectivorous farmland birds [7, 23], including house martins (*Delichon urbicum*) and barn swallows (*Hirundo rustica*). These species have experienced severe population declines across Europe for decades [24, 25], reflecting reductions in prey availability [26] and prompting changes in their red-list to more threatened categories in several European countries (e.g. [27, 28]. Both species suffer from the combined effects of land-use change and reduced food availability, which, for example, decrease individual fitness via adverse effects on adult and nestling body condition [29], nestling growth and brood size [30], fledgling success [31] and overall bird abundance [32].

House martins (hereafter HM) and Barn swallows (hereafter BS) occur sympatrically across most of Europe and frequently nest in close proximity. Most previous studies have analysed habitat selection in BS alone, rarely incorporating distance to the nest (but see [14]). BS generally avoid arable fields and prefer water, extensive grasslands and hedgerows for foraging [14, 33–35]. HM remain comparatively understudied, with existing evidence focusing on breeding-site and landscape associations [36, 37] rather than direct foraging habitat selection (but see [38]). The limited foraging evidence available suggests broadly similar habitat preferences to BS: selection for water bodies, urban areas, and extensively managed agricultural land and avoidance of intensively managed arable fields, forests, and orchards. Partial niche differentiation between the two species has been described along prey-size and air-space use axes: BSs pursue larger, faster flying insects close to the ground, whereas HMs hunt smaller, less mobile prey at higher altitudes and at longer distances from the colony [38–40]. Such vertical, trophic, and spatial differentiation could reduce direct overlap in resource use. The extent to which these species share or segregate space within intensively managed landscapes remains unknown, as no studies have jointly examined horizontal habitat selection in both species in relation to distance from the colony. Understanding how sympatric aerial insectivores simultaneously use space in intensively managed landscapes, particularly under ongoing insect decline, is essential for characterising their joint space use, with potential implications for coexistence and for conservation at field and landscape scales.

In this study, we used the ATLAS system (Advanced Tracking and Localisation of Animals in real-life Systems; [41, 42]) to fit behavioural state-dependent step-selection functions for BS and HM across an intensively managed agricultural landscape with low insect availability. During tracking, we simultaneously sampled insects with Malaise traps distributed across the study area. We tested how (i) land use in combination with distance to colony, (ii) proximity to water bodies, woody vegetation structures (e.g. hedgerows), and habitat edges, and (iii) insect‐prey metrics (abundance, biomass, taxon richness, body size) shape state-specific habitat use conditional on colony accessibility during commuting, foraging, and resting. Because step-selection functions estimate use relative to movement-conditioned availability, our inference targets movement-constrained habitat use rather than absolute habitat preference. We focused on whether distance from the colony modulates selection differently across behavioural states in each species. Grounded in central-place foraging theory, we made the following predictions:

### Distance-to-colony effects

Habitat selection would vary with distance from the colony, particularly during foraging and resting. With increasing distance, both species should increasingly select land-use types offering higher prey abundance and structural complexity (e.g. hedgerows, extensive grasslands) to compensate for travel costs through high returns [19]. Simultaneously, they should avoid structurally simple, prey-poor habitats (e.g. arable fields, intensive grasslands), with stronger effects in HM, which are expected to forage farther from the colony more frequently [38]. Resting should occur near structurally complex features providing shelter (e.g. woody vegetation), which should be selected more strongly farther from the colony, where prolonged foraging bouts make returning to the nest for rest impractical. During commuting, both species should align their movements with linear landscape elements such as hedgerows or field edges, which provide both navigation cues and energetic benefits via reduced flight costs on the leeward side [33]; because these benefits apply regardless of trip length, selection should be largely independent of distance to colony.

### Proximity to landscape features and prey effects

During foraging, both species should select for proximity to water bodies, woody vegetation structure, and structural edges (such as field edges, forest edges, etc.). These features harbour higher insect densities that spill into adjacent land-use types, decreasing with distance from the source [29, 43]. Both species were further expected to respond positively to local insect biomass or abundance during foraging. Insect taxonomic richness was expected to exert a similar or stronger influence, given evidence that diverse prey communities provide nutritional resources unavailable through abundance alone [9, 44] and stabilise food supply through asynchronous phenologies [45]. Finally, we predicted that HM would preferentially select smaller insect taxa than BS, consistent with their characteristic foraging at higher altitudes and slower flight speeds, which favour the interception of smaller, less mobile prey [39, 40].

## Material and methods

### Study area and land-use information

**Fig. 1:**
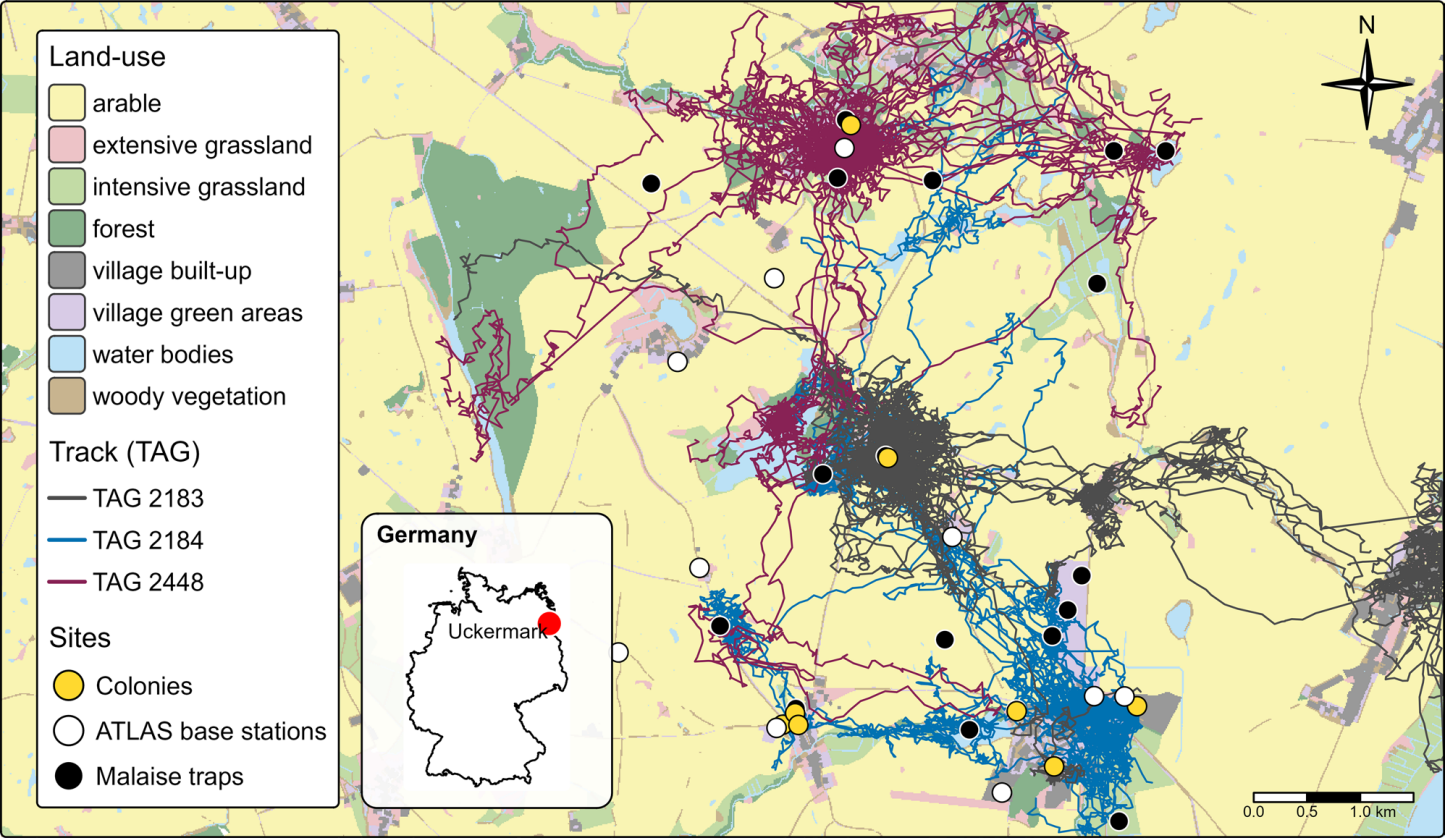
Land-use map, movement paths of three tagged individuals (two BS: TAG 2183, 2184, and one HM: TAG 2448), ATLAS base stations in 2023, bird colonies, and Malaise traps in the northwestern Uckermark, Germany. Dairy farm 1 is the northernmost colony, dairy farm 2 is the easternmost colony (see Additional file 2). CRS: UTM Zone 33 N (WGS84; EPSG:32633). The inset shows the location of the study area within Germany (red dot). For detailed visualisation of the land-use types, please see Fig. S1-2 and for Malaise trap placement please see Fig. S1-5

We studied hirundine movement behaviour in northeast Germany, ~ 100 km north of Berlin (53°35’N; 13°68’E, Fig. 1). The study area was characterised by large-scale, intensive farming with large crop fields (26 ± 29 ha, mean ± SD). Arable land covered 77% of the landscape, dominated by wheat, maize, and oilseed rape, interspersed with grasslands, villages, water bodies (small lakes, streams, ditches and kettle holes), small forests, and other woody vegetation structures, defined as linear or isolated tree and shrub stands [46]; see Additional file S1-1 for percent cover). Based on biotype maps [46], we created a raster (1 m resolution) using the R packages *sf* and *terra* [47, 48] to represent the above-mentioned land-use types (details in Additional file S1-2). We chose a 1 m resolution to preserve sharp boundaries between land-use types, particularly between arable land and adjacent vegetation, so that each location could be assigned to the correct land-use type. We buffered linear and point features (i.e., hedgerows, small streams, solitary trees) with a 7 m buffer to approximate their typical width (~ 14 m) and potential insect prey spillover. We also generated 1 m distance rasters for water bodies, woody vegetation, and structural edges.

### Animal tagging and tracking

Between 2019 and 2024, we deployed 406 ATLAS tags on 361 individuals (165 HM and 196 BS; Additional file S2). Breeding birds were captured mainly in May and June within each year (for details please see Additional file S2) using mist nets at two large dairy farms and several small private stables within villages (Fig. 1). Both species nested at the largest dairy farm; private stables hosted single-species colonies; at the second dairy farm, we mainly captured HM. We ringed, measured, and weighed the birds. We checked our morphological sexing success in 2023 against DNA test for 245 birds, where both species proved to be unreliable sexed (HM accuracy was 61%, BS 78%, M. Grabow, unpublished data), hence to maintain consistent analyses across species, sex was not considered further. This represents a limitation of our study, as sex and breeding role can influence movement, provisioning, and habitat use in aerial insectivores. We tagged BS weighing > 19 g and HM > 20 g with ~ 1 g ATLAS tags, which are part of a reverse-GPS system with a typical horizontal location error of about 5.7 m [49, 50]. In contrast to GPS, where tags receive signals from satellites and compute their own positions, ATLAS tags transmit signals that ground-based base stations trilaterate (i.e. trilateralization, a distance-based approach, as opposed to the direction-based triangulation) to determine the tag’s location. Our ATLAS system consisted of a network ranging between 8 and 11 base stations, depending on the year (Fig. 1). Tags transmitted signals to the ATLAS antenna until either the battery ran out or they fell off. We glued the tags to birds’ backs, between the wings, using Sauer surgical skin glue and Walker wig glue. The tags transmitted every 4–8 s (Additional file S2).

### Pre-processing steps and tag failures

For the analysis, we subsampled all data to an 8-second interval. We processed and filtered the raw data mainly following Gupte et al. [51] (Additional file S1-3) retaining only locations meeting our trilateralization precision threshold and individuals with sufficient tracking duration after the first three hours post-capture. Of the 406 tags, 158 (39%) failed or detached shortly after deployment, and 35 (9%) were excluded during filtering for not meeting these criteria (please see Additional file S2 for retention rates). The remaining 213 tags were attached to 204 individuals. We also excluded 16 tags from a 2019 pilot study, when birds were tagged mainly in late August/ early September, because central-place foraging was no longer assured. This yielded 197 tags on 188 individuals (101 HM, 87 BS) for analysis, with nine birds re-captured and re-tagged across years (Additional file S2).

### Behavioural state classification

We labelled each location into three behavioural states: stationary (resting, non-moving), commuting (direct, fast flight) and foraging (slower, less directed flight associated with prey search and capture). We did this by first classifying stationary behaviour using the *recurse* R-package [52], which quantifies residence time (here 200 s) within a user-defined radius (here 20 m) around each location (details in Additional file S1-4). Secondly, we classified all non-stationary locations into commuting and foraging using two-state hidden Markov models (HMM, *momentuHMM*, [53]), with gamma step-length and von Mises turning angle distributions. We used species, year and their interaction as covariates. To satisfy the assumption of regular time steps in HMM models, step lengths and turning angles were set to missing when temporal gaps exceeded 20 s, so that movement metrics were not calculated across large gaps (details in Additional file S1-4).

### Insect prey sampling

In 2023, we sampled insects concurrently with bird tracking using Malaise traps (following [54, 55]) to characterise insect community composition across land-use types. Traps operated during 23–30 May and 26 June-3 July. We distributed 16 Malaise traps across the study area (Additional file S1-5), sampling the following land-use types: arable (3 traps in wheat), grasslands (3 traps in intensively used grasslands), villages (4 traps in gardens, one more trap to account for higher within-habitat heterogeneity), woody vegetation structures (3 traps, inside hedges), and close-to-water-bodies (3 traps next to water bodies). Trap locations were selected to provide stratified coverage of the five dominant land-use types in the study area, with opportunistic placement (i.e. far away from crop/grassland borders; only in hedges running from north to south) within each habitat to ensure spatial spread. Samples were stored in 99% ethanol at 4 °C. In the lab, insects were identified to order (Diptera split into Brachycera and Nematocera), body size was measured, and dry mass was obtained after drying and weighing.

### Habitat selection

We removed all locations between civil dusk and dawn (sun angle 6° below the horizon), when hirundines are generally inactive, as we were interested in the active-period habitat selection, including daytime resting behaviour. The remaining data were split into commuting, foraging, and stationary subsets. For commuting and foraging, we generated 10 random “available” steps for each observed “used” step using step-selection functions [56]. To avoid sampling the same habitat repeatedly due to very short steps, which would generate available locations within the same land-use class as the used location and bias selection coefficients toward zero, we thinned fixes to a 64s interval before random-step generation. Because gaps occurred (e.g. birds inside buildings, or when excluding other behavioural states), we clustered data into bursts of regular times using the R package *amt* [57], and removed bursts with fewer than three steps. We generated random steps from gamma (step length) and von Mises (turning angle) distributions, fitted separately for each species and behavioural state (commuting, foraging). For the stationary subset, we first calculated the centroid location of each stationary event (the x and y medians of the locations scattered around the birds’ stationary position). Then we generated 10 random locations within a 500 m radius (between the 95th and 99th percentile of step lengths during foraging and commuting flight pooled across species) of the last location to represent available resting sites. For all used and available locations, we extracted land-use type at the exact location (point extraction) from the 1 m raster, along with distance to the nearest structural edge, water body, and linear or isolated woody vegetation structure from the rasters, and calculated Euclidean distance to the colony.

### Statistical analyses

To test whether HM and BS differed in distance from the colony, we aggregated high-frequency locations to daily summaries per individual and calculated mean and maximum daily displacement from the colony. We modelled these separately using generalized linear mixed-effects models with Gamma errors and log link (*glmmTMB*; [58]). Species was entered as a fixed effect, and individual as a random intercept. Throughout the study, we checked the model assumptions using the package *DHARMa* [59] and discarded variables with high multicollinearity (r < |0.6|, [60]).

The insect data was first analysed alone (i.e. without including the movement data). We assessed multicollinearity among biomass, abundance, taxonomic richness, and mean body size. Biomass and abundance were strongly correlated (*r* = 0.75), so we retained abundance and omitted biomass. To test how the single insect metrics varied with land-use type, we ran separate mixed-effects models (Gaussian errors) for abundance, richness, and body size. In each model we used the respective insect metric as the response, while land-use type and month (May, Jun), as well as their interaction, were fixed effects, and trap ID and date were random intercepts. To test how the individual insect metrics responded to the trap’s distance to the nearest structural edge, we fitted a parallel set of mixed-effects models (Gaussian errors) for abundance, richness, and body size. The fixed effect in each model was distance to the nearest structural edge and random intercepts were trap ID and date. In a second step we combinedly analysed insect and movement data to investigate the influence of insect community on bird habitat selection, which is described below.

Habitat selection for commuting, foraging, and resting was analysed separately using mixed-effects Poisson models with a log link and a stratum-specific random intercept (each used step and its associated available points), which are likelihood-equivalent to conditional logistic mixed models [61]. The binary response indicated used (1) versus available (0) steps. Fixed effects were species (HM, BS), tagging location (colony), distance to colony, and feature proximity (distance to water, woody vegetation, and habitat edge), with feature proximity calculated regardless of the current distance to colony. All continuous covariates were centred and scaled. Interaction terms included a three-way interaction between land-use type, distance to colony, and species, and two-way interactions between species and each feature-proximity variable. Since the birds have to return to their colony, the distance to the colony was included with second-order orthogonal polynomials. For the proximity variables, we compared models with and without quadratic terms using AIC, retaining quadratic terms for proximity to wood and water, while edge was modelled linearly. Arable land, the most prevalent land-use type, was used as the reference category. Individuals without used locations in the reference category (or other critical categories) were excluded, resulting in 138 birds for commuting, 170 for foraging, and 134 for resting. Further, following Muff et al. [61], we fixed the stratum-level random-intercept variance at a large value (10^6^) to avoid shrinkage towards zero. At the individual level (bird ID), we included uncorrelated random slopes for land-use type to allow individual-specific selection gradients. Because HM and BS were unevenly distributed across colonies (see Additional file S2), the species differences in habitat selection could in principle reflect colony-level landscape availability rather than species-level selection. We therefore performed two sensitivity / confound checks. First, we compared the proportional cover of each land-use type in four bands around each colony (0–500 m, 500–1000 m, 1000–2000 m, 2000–3000 m), capturing the bird’s typical foraging range. Second, we refitted the foraging SSF using only data from the single large dairy farm where both species co-occurred in substantial numbers (42 HM and 46 BS individuals, 94 tags total).

Because insects were sampled only during two 8-day campaigns in 2023, we fitted step-selection models for foraging locations recorded during those same 16 days, ensuring temporal matching between bird positions and the sampled insect communities. Insect metrics from each Malaise trap were assigned to the land-use type in which the trap was located, with a separate value for each sampling day (Additional file S1-6). This day-by-day assignment provides temporal variation in insect communities that the categorical land-use variable cannot capture, but within a given day all cells of the same land-use type share the same insect metric value, so the mapped predictors are not spatially independent of land-use type. We therefore interpret the contrast between the land-use SSF and the mapped insect SSFs as a comparison of alternative habitat parameterisations that differ in their temporal resolution, rather than a test of spatial within-habitat prey effects. We used the same conditional logistic mixed-effects framework as above, including land-use type as a predictor, then replacing land-use by each insect metric (abundance, taxonomic richness, mean body size) in turn to assess their relative explanatory power. To capture joint variation in insect metrics, we also performed a principal component analysis (PCA) on all four metrics and fitted models using PC1 or PC2 as composite predictors.

In the results section, we reported *log*-Relative Selection Strength (log-RSS) with 95% confidence intervals. The log-RSS values are interpreted relative to the reference category (arable land) and to model-defined availability (the random “available” steps generated for each “used” step), where positive logRSS values indicate selection above the reference category, and negative values indicate selection below it. Confidence intervals for continuous predictors were derived via parametric bootstrap of the fixed-effects variance–covariance matrix [60]. All statistical analyses were conducted using R 4.5.0 [62].

## Results

The 197 tags deployed between 2020 and 2024 recorded, on average, 18,502 ± 18,225 (mean ± SD, range 1177–99814) locations over 3.7 ± 3.0 days (range 0.54–12.89 days), corresponding to 3,801 ± 2,628 daily locations per ATLAS-tracked bird. Observed mean and maximum daily displacement from the colony were 573 ± 310 m and 2,805 ± 1,389 m for HM, and 448 ± 667 m and 1,855 ± 1,149 m for BS, respectively. Our models supported species differences in displacement: the BS/HM mean-distance ratio was 0.67 (95% CI 0.57–0.78) and the max-distance ratio was 0.64 (95% CI 0.56–0.74), implying that BS had on average 33% shorter displacement distances than HM (Additional file S1-7).

The 16 Malaise traps collected a total of 102,267 insects across the study area and sampling period (Additional file S1-8). The most abundant (sub-)orders were Brachycera (57,437 individuals), Nematocera (25,012), Hymenoptera (12,805), Hemiptera (3,506), Coleoptera (2,155), and Lepidoptera (670; Additional file S1-9). Insect biomass averaged 1.4 ± 1.3 g per trap and day, with 401 ± 340 individuals per trap and day (range 15 to 2,252 individuals), taxonomic richness of 6.3 ± 1.5 taxa, and mean insect body size of 3.6 ± 0.8 mm.

### Insect metrics in relation to land-use types

**Fig. 2:**
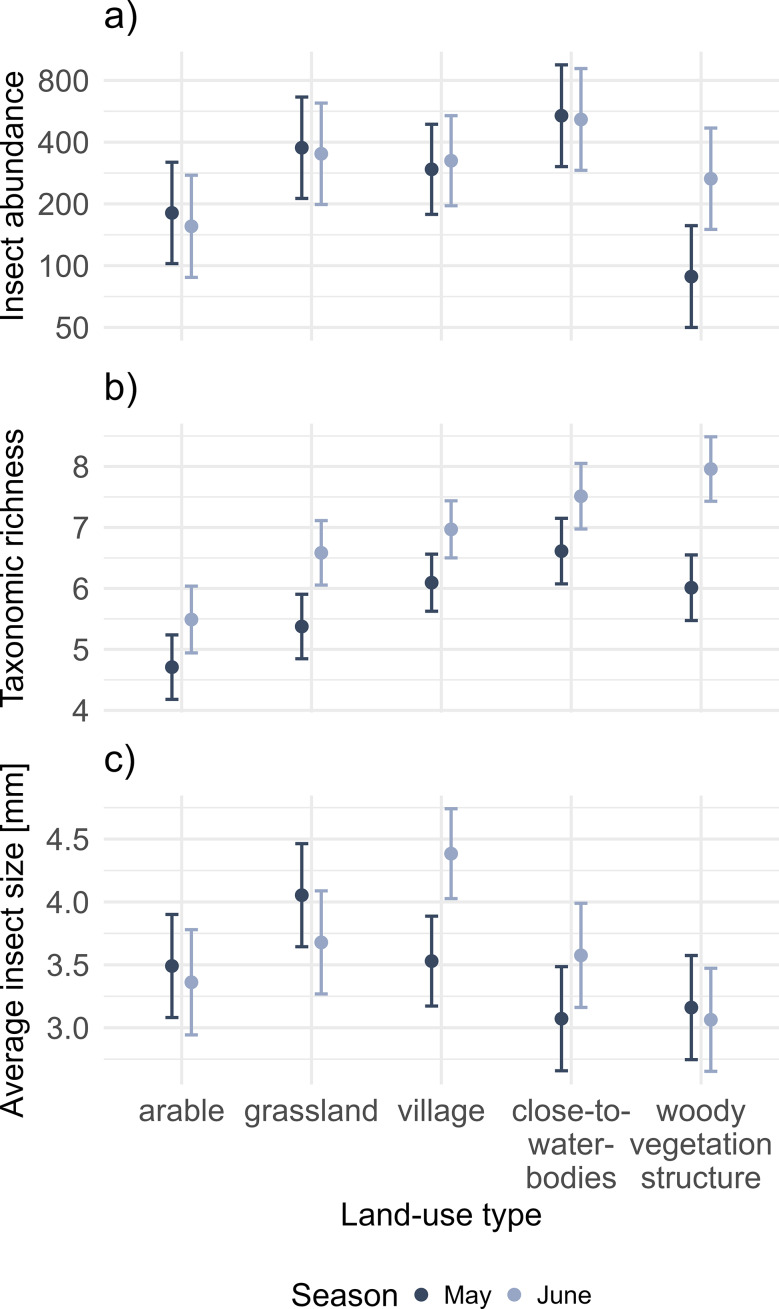
Predicted (**a**) abundance, (**b**) taxonomic richness, (**c**) mean body size (mm) of insects across land-use types and month from generalized linear mixed models. Points are model-predicted means by land-use and month; vertical bars indicate 95% CIs

Prey abundance was higher in grasslands, villages, and near water bodies than in arable land, while woody vegetation did not differ significantly from arable fields (Fig. 2, Additional file S1-10). Taxonomic richness was higher in all habitats than in arable fields, with the strongest positive effect in woody vegetation, but also near water bodies and in villages. Mean prey size was larger only in villages. Insect abundance in woody vegetation was significantly higher in June than in May, while taxonomic richness was higher in June for all land-use types, with a more pronounced increase in woody vegetation, consistent with phenological accumulation of late-emerging woodland-specialist insects. Insect sizes were larger in June in villages and near water bodies. Distance to the nearest structural edge did not affect prey abundance or mean body size, but instead taxonomic richness declined by about 11% (0.65 taxa) per 100 m from field edges.

### Land-use selection relative to arable fields and dependent on behavioural state and central-place distance

**Fig. 3:**
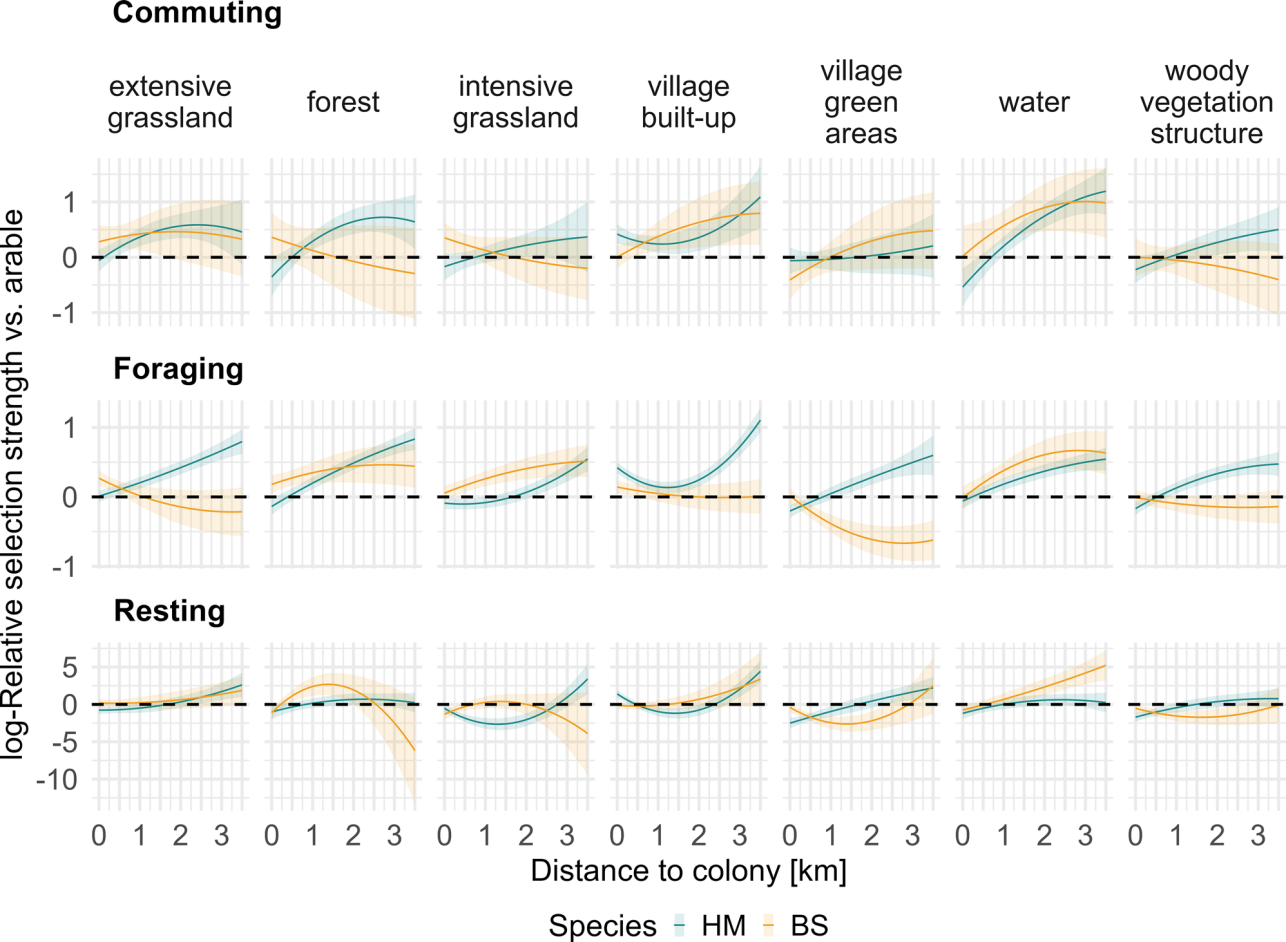
The effects of the interaction between land-use type and distance from their colony on the logarithmic Relative Selection Strength (log-RSS) for HM (teal) and BS (gold) during commuting (upper row), foraging (middle row), and resting (lower row). Values above the dashed horizontal line indicate selection regarding the reference category (arable), while values below the line show avoidance. The ribbons represent the 95% confidence intervals. Resting over water bodies occurred mainly on waterlogged trees and overhanging branches

Both species spent similar proportions of time commuting (~ 3 ± 3%), foraging (~ 64 ± 19%), and resting (~ 33 ± 20%, Fig. S1-4.1). Across behavioural states, HM and BS exhibited distance-dependent habitat selection relative to arable land (Fig. 3, Additional file S1-11). Selection patterns reflected species-specific differences rather than variation in local landscape structure: landscape composition was broadly consistent across colonies (Additional file S1-12.1), and results were qualitatively unchanged when restricting the analysis to a single large colony where both species co-occurred (Additional file S1-12.2).

Throughout, log-RSS values represent selection or avoidance relative to arable land (the reference category). All log-RSS point estimates reported below are accompanied by 95% confidence intervals in Table S1-11.1 (full estimates) and in Fig. 3 (visual representation). During commuting, both species selected villages and water bodies more strongly at greater distances from the colony, with the distance effect particularly pronounced for water bodies (logRSS for HM increased from − 0.15 at 500 m to 0.75 at 2000 m and for BS from 0.32 to 0.90 across the same distance range). Commuting HM generally showed increasing selection for structurally complex habitats with increasing distance from the colony, particularly for extensive grasslands, forests, and woody vegetation structures, whereas commuting BS showed mainly neutral distance responses across most land-use types (Fig. 3).

During foraging, both species mainly avoided arable land and selected structurally complex habitats with higher mapped insect abundance and/or taxonomic richness. These effects strengthened with distance from the colony and were mostly steeper for HM. Both species increasingly selected forests (logRSS for HM increased from 0.03 at 500 m to 0.49 at 2000 m and for BS from 0.27 to 0.44) and water bodies (logRSS for HM increased from 0.07 at 500 m to 0.37 at 2000 m and for BS from 0.20 to 0.61) with distance from the colony, though BS selection strength for forests plateaued at ~ 1.5 km from the colony. Extensive grasslands and villages were selected more strongly near colonies by both species, but HM maintained this selection also farther away (logRSS for HM increased from 0.10 at 500 m to 0.43 at 2000 m); while BS showed neither selection nor avoidance at greater distances from the colony. BS selected intensive grasslands with increasing distance to the colony, while HM avoided intensive grasslands up until ~ 2 km from the colony and then switched to selection. HM avoided green spaces in villages near colonies but selected them in more distant villages. BS, on the other hand, selected villages and were neutral to green areas, around colonies, and avoided both habitats at greater distances. During resting, both species showed mainly increasing selection with increasing distance from their colonies, indicating greater use of distant resting sites within the available range. BS showed a stronger propensity to rest in forests than HM, but both species used forests as resting places (logRSS for HM increased from − 0.35 at 500 m to 0.15 at 1000 m and for BS from 1.11 to 2.38 across the same distance range).

### Selection for proximity to structural edges, water bodies, and woody vegetation

Selection for proximity to habitat features was largely consistent across behavioural states (Additional file S1-13), so we report only the combined results here. Both species selected for proximity to structural edges, although effect sizes were generally small. HM selected strongly for close proximity to water bodies (up to ~ 130 m), whereas BS preferred intermediate distances to water. Both species selected for proximity to woody vegetation structures, but BS did so more strongly than HM.

### Relative importance of mapped insect community metrics

Comparing the mapped insect models to the land-use model showed that the model containing the land-use type was by far the best (∆ AIC = 1021, Additional file S1-14), followed by taxonomic richness, PC2, PC1, median insect size, and abundance. PC2 mainly captured variation in taxonomic richness and median insect body size, whereas PC1 was dominated by abundance and biomass. Insect taxonomic richness increased habitat selection relative to the median (6 taxa), mainly for HM, with BS showing wide confidence intervals overlapping zero. HM selected to forage in areas of high mapped insect richness close to and far from colonies, whereas BS showed the strongest selection for high mapped insect richness at intermediate distances from colonies (Fig. S1-14.2). Median insect body size did not significantly improve model fit, and selection coefficients for prey size were not significantly different from zero for either species (Table S1-10.2).

## Discussion

Our study revealed that habitat selection in house martins (HM) and barn swallows (BS) depended on land-use type, behavioural state, and distance from the colony, consistent with predictions from central-place foraging theory. HM foraged significantly farther from colonies than BS, likely reflecting energetic species-specific trade-offs between travel distance and prey type (e.g. aquatic insects from distant water bodies). HM also showed stronger distance-dependent selection for structurally complex habitats than BS, and the two species used those habitats differently: HM selected more strongly for proximity to water bodies, whereas BS selected more strongly for areas close to woody vegetation structures. These contrasting patterns indicate differential space use between the two species under shared central-place constraints, which may have implications for interspecific competition.

### Behavioural state differences

While habitat selection is known to vary with behavioural state because different actions require different resources [63], we extend this concept for central-place foragers by demonstrating that habitat selection is distance- and species-dependent across behaviours. For example, HM commuted over forests primarily at greater distances from the colony, while BS did so mainly at close distances. During foraging over forests, the distance dependence was greater for HM than for BS, whereas during resting, the reverse pattern occurred. Both species also exhibited distance-dependent selection for extensive grasslands and water bodies during commuting and foraging, habitats previously identified as key foraging areas for hirundines [38, 64]. Our results show that these open habitats are also important for commuting and resting, with their use varying by species and distance from the colony.

As predicted, resting behaviour was concentrated in structurally rich habitats, especially farther from the colony, where both species rested in extensive grasslands (perching on long grasses and/or power lines, personal observation) or over water bodies (using waterlogged trees, overhanging branches, and/or reeds) during prolonged foraging bouts. Thus, our results illustrate that combining distance to the colony [65] and behavioural state [66] can reveal species-specific habitat-selection patterns that would remain obscured if these dimensions were ignored.

As predicted, both species selected for proximity to linear and structural landscape elements (e.g. edges and woody vegetation). Although we expected these features to be most important during commuting as navigational aids, selection for feature proximity was broadly similar across behavioural states, suggesting a general reliance on structural cues and/or foraging opportunities associated with edges and woody vegetation throughout daily movement. From a central-place perspective, such linear elements may still facilitate efficient travel between colonies and profitable patches (e.g. orientation cues or reduced flight costs by using the lee side of windbreakers; Evans, 2003), but our data indicate that these benefits are not restricted to commuting alone.

### Species differences

Different species that share a central-place and similar resources may co-exist by differentiating horizontally [12], vertically [67], temporally [68], or trophically [69]. Despite previously described vertical and trophic partial niche differentiation in our two focal bird species, we also found pronounced differences in horizontal habitat selection between foraging HM and BS. However, differences in habitat selection do not necessarily imply complete spatial segregation; both species can still use the same habitat patches simultaneously, but with different probabilities [68]. When such overlap occurs under resource scarcity, HM and BS may additionally rely on vertical and trophic niche differentiation which could reduce competition.

Especially HM, the species operating farther from the colony, showed stronger selection with increasing distance across all habitat types except arable fields (which had the lowest insect abundance and fewest insect taxa). Comparable distance-dependent patterns have also been observed in another aerial insectivore, the Tree swallow (*Tachycineta bicolor*, [22]). However, in simplified landscapes, increasing travel costs with distance from the colony are also expected to constrain habitat choice, such that individuals may use habitats where prey abundance/diversity remains above a minimum profitability threshold, as predicted by central-place foraging and sequential search theory [18, 70]. In our system, intensively managed grasslands often meet this threshold because insect abundances are comparable to those in villages and water-associated sites. However, their apparent attractiveness may also reflect profitable microhabitats within or bordering intensively managed grasslands (e.g. hedgerows and water bodies), rather than the grassland fields per se—supporting the idea that hirundines respond to fine-scale structural features within broad land-use categories. This interpretation also aligns with barn swallows, which selected intensive grasslands consistently across the distance gradient, plausibly because they concentrated foraging along hedgerows adjacent to these fields [33].

During foraging, both species selected for forests, likely reflecting use of the airspace above the canopy rather than the forest interior (personal observation and unpublished data). This is compatible with the observation that hirundines avoid foraging within forests, hunting instead in open airspace where the pursuit of fast prey is unimpeded by vegetation [33, 36, 71]. Forest selection may, however, depend on landscape composition [72]: in our study area, forests covered only 2% of the landscape, and open habitats were widely available, suggesting active selection for canopy prey. This interpretation accords with evidence that canopy airspace can support higher prey abundance or richer prey communities than open agricultural land [8].

### Prey-related mechanisms

Foraging theory predicts that consumers should track spatial and temporal peaks in resource availability, concentrating activity in habitat patches where expected energy gain is highest [18, 73]. Nutritional ecology further suggests that animals should preferentially use habitats that provide not only abundant but also nutritionally suitable or diverse prey [9, 74]. In line with these predictions, both our species selected for proximity to water bodies (HM for close proximity, BS for intermediate proximity), woody vegetation, and field edges—structurally complex, prey-rich, and diverse habitats supporting spillover of insect prey [29, 43]. HM’s selection for close proximity to water aligns with their exploitation of small, emergent aquatic insects rich in polyunsaturated fatty acids [75, 76]), an essential dietary compound for aerial insectivores [9]. BS, by contrast, mainly target larger, faster terrestrial insects at intermediate distances from water [77, 78], and are less dependent on aquatic prey because they can maintain high levels of long-chain omega-3 fatty acids via efficient endogenous conversion from terrestrial sources [79]. Thus, HM’s greater reliance on nutrient-rich aquatic prey may compel them to range farther from the colony than BS, with these nutritional requirements helping to structure horizontal niche differentiation between the species.

For our mapped insect habitat selection models, we expected both hirundines to track patterns in the sampled insect community (prey diversity or at least prey abundance), as shown for bats tracking ephemeral insect prey swarms in agricultural landscapes [80] and for insectivorous birds that shorten foraging distances and increase colony visit frequency on days with high aerial insect abundance [13]. However, in our 2023 study, the model that included land-use type outperformed all insect-based models, suggesting that landscape structure remains a stronger determinant of foraging-site choice than short-term prey fluctuations at our sampling scale. We see two non-exclusive explanations. First (ecological): birds may exhibit site fidelity, repeatedly visiting familiar land-use elements known to yield insects, rather than re-optimising to the daily prey field [81, 82]. Such site fidelity can increase overall foraging efficiency [82] and is favoured when prey is predictably patchy or when exploratory foraging is costly, as in industrialized farmland [81]. Second (methodological): although our mapped insect predictors captured day-to-day variation in insect communities across the sampling period, they could not resolve spatial variation within habitat classes — all cells of the same land-use type shared the same insect value within a given day. The mapped insect SSFs therefore did not account for spatial fine-scale variation in prey availability, which likely contributed to the stronger performance of the land-use model. Furthermore, our Malaise traps sampled insect communities at trap level, which may not fully reflect the aerial prey composition at the foraging altitudes used by hirundines [83]. Nevertheless, among the insect metric models, both species (HM more strongly than BS) responded more to mapped insect taxonomic richness than to mapped abundance during our May-June 2023 sampling period. Two mechanisms could explain this pattern: diverse prey communities can provide complementary nutritional resources beyond what abundance alone captures [9, 44], and asynchronous phenologies among arthropod species can buffer food availability against species-specific fluctuations [45]. Our single-season sampling design cannot distinguish between these mechanisms. Contrary to our prediction, we did not detect stronger selection by HM for smaller insect size, possibly due to insufficient resolution in the prey data.

### Coexistence mechanisms and future directions

In our system, the two species optimise the central-place trade-off differently: HM incur higher travel costs but gain access to prey-rich, structurally diverse patches farther from colonies, while BS minimise travel distances by exploiting profitable sites closer to the nest. The resulting distance-dependent divergence in space use is consistent with stabilizing mechanisms that have been proposed for sympatric central-place foragers in energetically constrained landscapes [12, 84] — patterns that may become increasingly important as agricultural landscapes lose even more resources, e.g. with ongoing insect declines [85]. The observed species differences may also reflect non-competitive mechanisms (i.e. differences in body size, flight behaviour, or alternative travel-cost strategies). Furthermore, sympatric aerial insectivores can also converge on profitable resources rather than partition them [14]. To clarify coexistence mechanisms when lower trophic levels are depleted, studying both species in sympatry and allopatry will be crucial, with allopatric systems providing baseline space-use data in the absence of interspecific competition [86]. Functional habitat selection, such as seasonal or even hourly differences in habitat use, might further support niche differentiation [87, 88]. Although we accounted for individual variation statistically, we did not explicitly address whether individuals under higher feeding pressure (e.g. larger broods) are forced to behave differently. Incorporating these three-dimensional foraging decisions within a central-place framework may therefore help unify spatial and trophic partitioning into a single mechanistic understanding of coexistence.

### Upstream constraints from colony settlement

Colony location itself is the outcome of prior breeding-site attraction and nest-site selection. House martins and barn swallows have distinct nest-site preferences shaped by nesting substrate, building configuration and social information [37, 89–91]. Furthermore, both species show strong site fidelity, with adults frequently returning to previous breeding sites or natal colonies [92, 93]. Hence, the colonies we sampled represent historical settlement decisions. Once established, these locations constrain the spatial domain available to subsequent foraging, so the patterns we describe should be interpreted as conditional on those settlement processes rather than as independent of them. Therefore, the foraging patterns reflect species-specific responses to habitat given historically fixed central places [19, 20].

### Management implications

Our findings identify several habitats that disproportionately support foraging and resting in HM and BS within agricultural landscapes: water bodies, woody vegetation (including hedgerows and tree stands), forests, extensively managed grasslands, and green areas in villages. We therefore recommend management actions targeting each. For water bodies, conserving existing sites and re-wetting drained habitats would maintain or increase water availability. For woody vegetation, reducing crop field size by lining new borders with hedgerows and tree stands would benefit not only HM and BS but also insects, birds, and other vertebrates [94–96]. For forests, even the small area present in central European agricultural landscapes (about 2% in our study area) provides important resources for hirundines and should be protected from further fragmentation. For grasslands, extensive management with partial or rotational rather than uniform synchronous mowing would maintain refuge habitats and ensure continuous prey availability for breeding swallows. For green areas in villages, increasing the cover of native flowering plants would support foraging in built-up environments. Together, these measures can help maintain diverse, insect-rich foraging landscapes and support resilient swallow populations.

## Supplementary Information

Below is the link to the electronic supplementary material.

Supplementary Material 1

Supplementary Material 2

## Data Availability

The bird movement data is available, upon request, via movebank (Movebank ID: 3053965481). The insect dataset and the study underlying GIS information are available from the corresponding author on reasonable request.
