# Supplementary material for: Distance to central-place drives species-specific habitat selection in sympatric insectivorous birds
Source: Mov Ecol. 2026 Jul 3;14:49. doi: 10.1186/s40462-026-00676-4 (PMC13339423; doi:10.1186/s40462-026-00676-4)
Supplement: Supplementary file 1 — Supplementary Material 1 [file 40462_2026_676_MOESM1_ESM.docx]

# Supplementary material S1

## S1-1. Land-use types as percent cover in study area

**Table S1-1:** Percentages of the land-use types within the extent of the study area in northeast Germany in a typical agricultural landscape used for intensive farming.

| **Land-use type** | **Percent cover in study area** |
| --- | --- |
| arable | 77 |
| intensive grasslands | 6 |
| villages | 4 |
| water bodies and bogs | 4 |
| woody vegetation structures | 3 |
| extensive grasslands | 2 |
| forest | 2 |
| green areas in villages | 2 |

## S1-2. Land-use type categorization and raster creation

Our land-use and proximity to feature (structural edge, water bodies and woody vegetation) rasters were based on colour-infrared biotype (CIR) maps from 2009 (LfU Brandenburg 2009. CIR (colour-infrared)-Biotope types 2009. www.govdata.de/dl-de/by-2-0). We used the classification scheme from the CIR maps in most cases and manually adjusted the classification for some polygons that were outdated (Fig. S1-2). One area, for example, was classified as “storage area” and would have fallen into the category “village built-up” but was actually an extensive grassland – heavily used by foraging hirundines from the adjacent colonies. On the other hand, some grasslands that were classified as “extensive” in 2009, were used either intensively or even as arable fields during the course of our study. Furthermore, three artificial water bodies close to dairy farm 2, that hold no ecological value, were reclassified to represent the surrounding land-use category because they would have biased the analysis towards an avoidance of water bodies, but the reason for the avoidance would have solely been based on their artificiality.

##
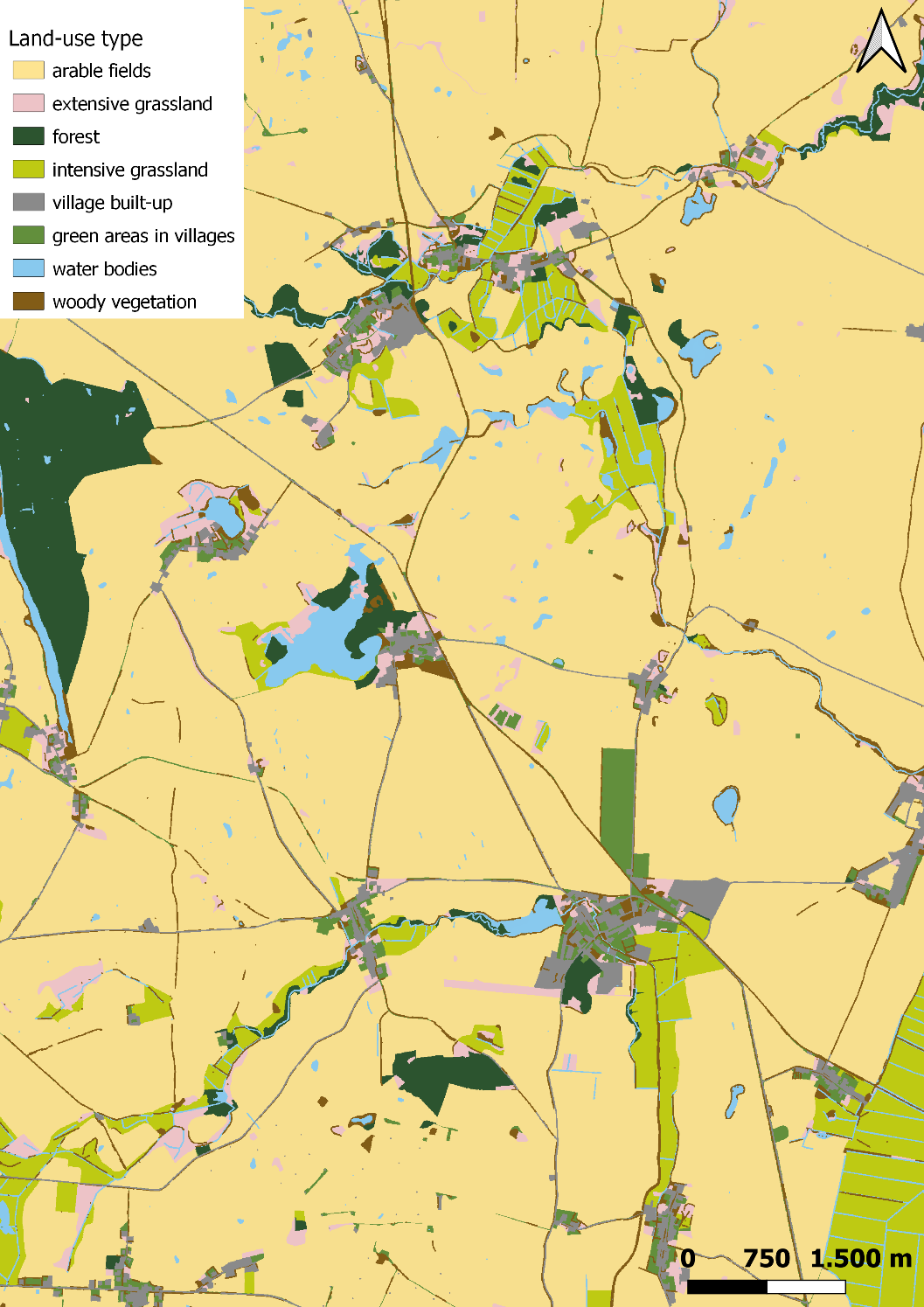


**Fig. S1-2**: Land-use map of the study area in the Northwest Uckermark, Germany. CRS: UTM Zone 33N (WGS84; EPSG:32633).

## S1-3. Raw data processing

We first deleted tags with fewer than 80 data points. Then we reduce location errors by filtering the raw data using a three-level confidence rank method based on the number of ATLAS antenna that received the signal and the standard deviation of the systems accuracy for each location (unpublished code provided by the ATLAS development team, 2022). For each location, this function 1) computes a horizontal precision metric (stdVarXY) from the variance–covariance matrix, 2) counts the number of base stations (NBS) involved in multilateration, and 3) runs two passes (forward + backward) through the time-ordered data to propagate confidence labels along spatially contiguous segments. We used the following parameters for the confidence ranking: maximum linking speed of v = 40 m s⁻¹ (conectedVel), precision threshold of stdVarXY < 80 m (stdlim), ≥ 7 receivers for immediate high confidence (2) (minNBSforConf2), ≥ 4 receivers for potential medium confidence (1) (minNBSforConf1), and the command to upgrade to high confidence (2) when at least 5 consecutive medium-confidence fixes occur within a spatial radius (the radius is calculated by multiplying the maximum linking speed and the nominal sampling interval (8 or 4 sec), resulting in radii of 320 m or 160 m. All fixes labelled low confidence were discarded; only medium and high confidence fixes were retained. After the confidence ranking and filtering, we continued the filtering process according to standard ATLAS filtering procedures (Gupte et al., 2022). We deleted all localizations whose variance-covariance standard deviation exceeded the 95th percentile of the given individual. Here, we furthermore distinguished between coordinates inside and outside the ATLAS antenna area, as locations farther away from the polygon created by the antennas usually display larger variances. After the variance filter we applied a speed filter (threshold value 20 m/s²) and a distance filter (threshold value 500 m), in which two sequential positions undergo a reality check. If the second position is only reachable from the first location using a speed exceeding the threshold, it is deleted. The same concept applies to the distance filter. After filtering the data we regularized the time series manually using the R-package *data.table* (Dowle et al., 2019). Subsequently we interpolated missing locations by using the simulate() function from the *ctmm* R-package (Calabrese et al., 2016). However, we only simulated data for time gaps smaller than 200 sec, which happens e.g. when the hirundines forage close to the ground and the tag signal cannot reach the antenna. To account for larger gaps, we divided the data set into location bursts, ensuring that a new burst begins after each gap of more than 200 sec.

## S1-4 Behavioural classification details

For the recurse analysis we considered a location as stationary, when the individual stayed for at least 200 sec inside a 20 m radius around the focal location. We decided to use a combination of the recurse analysis and a 2-state hidden Markov model (HMM) with species as a covariate, for the behaviour classification, as visual examination showed a slightly better fit of resting locations for the recurse analysis instead of a 3-state HMM. However, the HMM models assume a regular time series, but our data had gaps, due to excluding the stationary data, but also due to missing locations. Therefore, we flagged step lengths and turning angles as missing when temporal gaps exceeded 20 seconds, as movement metrics could not be reliably calculated across these gaps. We then deleted those data points, as the classification into different behaviour types was not possible for them. We subsequently quantified the proportion of data lost during this process together with lost data during random_steps() in the R package *amt*, that needs at least three locations to form a random step, as described in the section “Habitat selection”. For both processes together, we lost 1.8% of the movement data. Our HMM fitted gamma distributions for step lengths and von Mises distributions for turning angles, with commuting behaviour characterized by longer, more directional movements and foraging behaviour by shorter, more tortuous movements, i.e. we used different step lengths mean and SD for the two states (commuting and foraging) and also different turning angle means and concentrations. Furthermore, we used step bounds and angle bounds (including the step length SD and the angle concentration) to construct our HMM, we also included species and year (and their interaction) as covariates. The resulting transition probability matrix (based on mean covariate values) can be found in Table S1-4, and the final behavioural classification can be seen in Fig. S1-4.1. We also provide labelled example tracks (Fig. S1-4.2). All threshold values were initially motivated by a combination of biological and methodological considerations: 200 s residency time was chosen to distinguish sustained perching/resting events from brief flight pauses; 20 m buffer radius corresponds to approximately three to four times the typical ATLAS location error (~5.7 m), ensuring that a genuinely stationary bird remains within the buffer despite location noise; and the 20 s maximum allowed gap for HMM input covers two consecutive missing fixes at our 8 s native sampling rate. The initially chosen values were subsequently refined by testing a range of alternative values in both directions, with visual inspection of the resulting state classifications used to assess robustness and to select values producing biologically sensible outputs.

**Table S1-4**: Transition probability matrix from the HMM, based on mean covariate values, for foraging and commuting.

|  | Foraging | Commuting |
| --- | --- | --- |
| Foraging | 0.985 | 0.015 |
| Commuting | 0.163 | 0.837 |


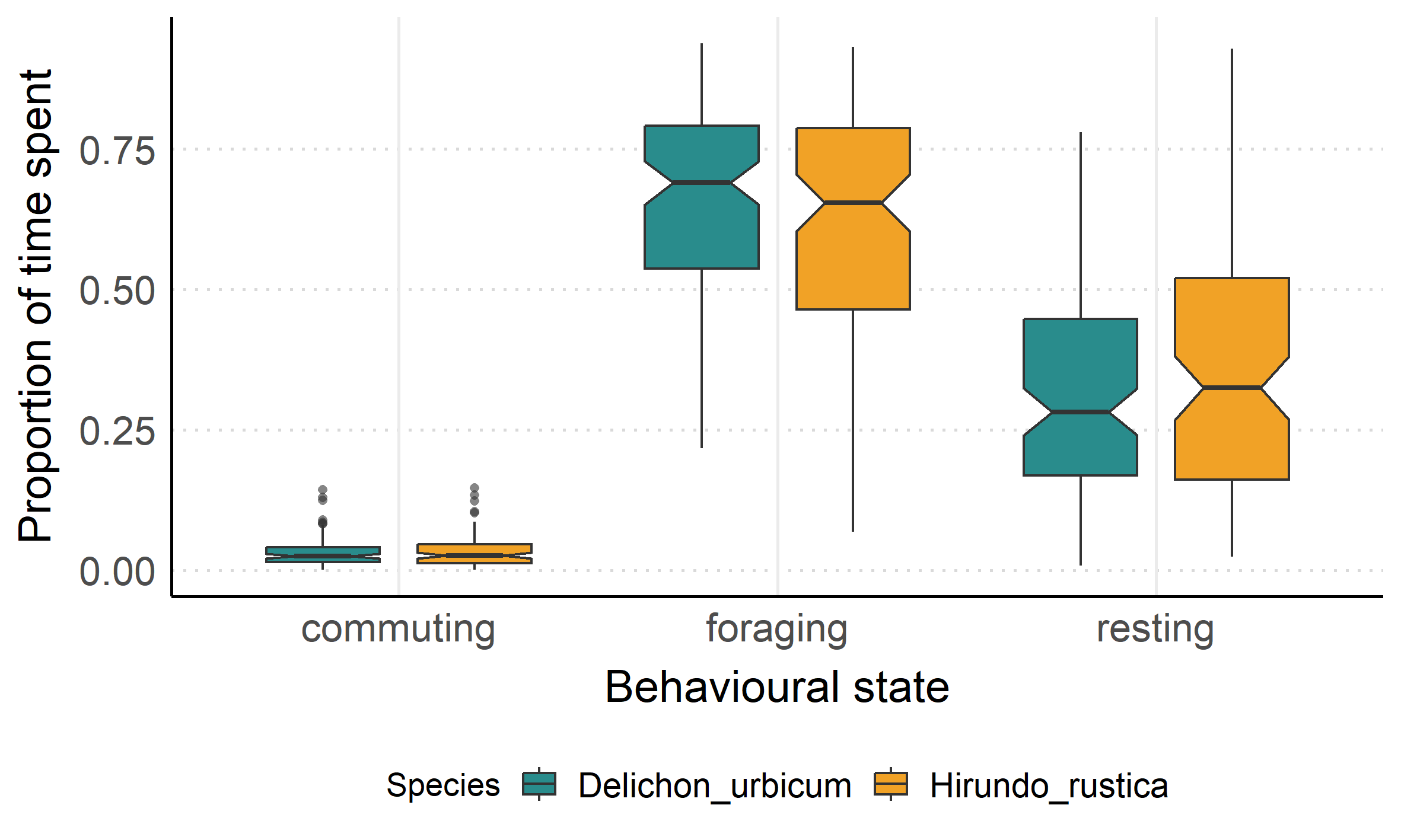


**Fig. S1-4.1.** Proportion of time HM and BS spent in each of the three behavioural states (commuting, foraging and resting).

## S1-5 Map of malaise traps within the study area


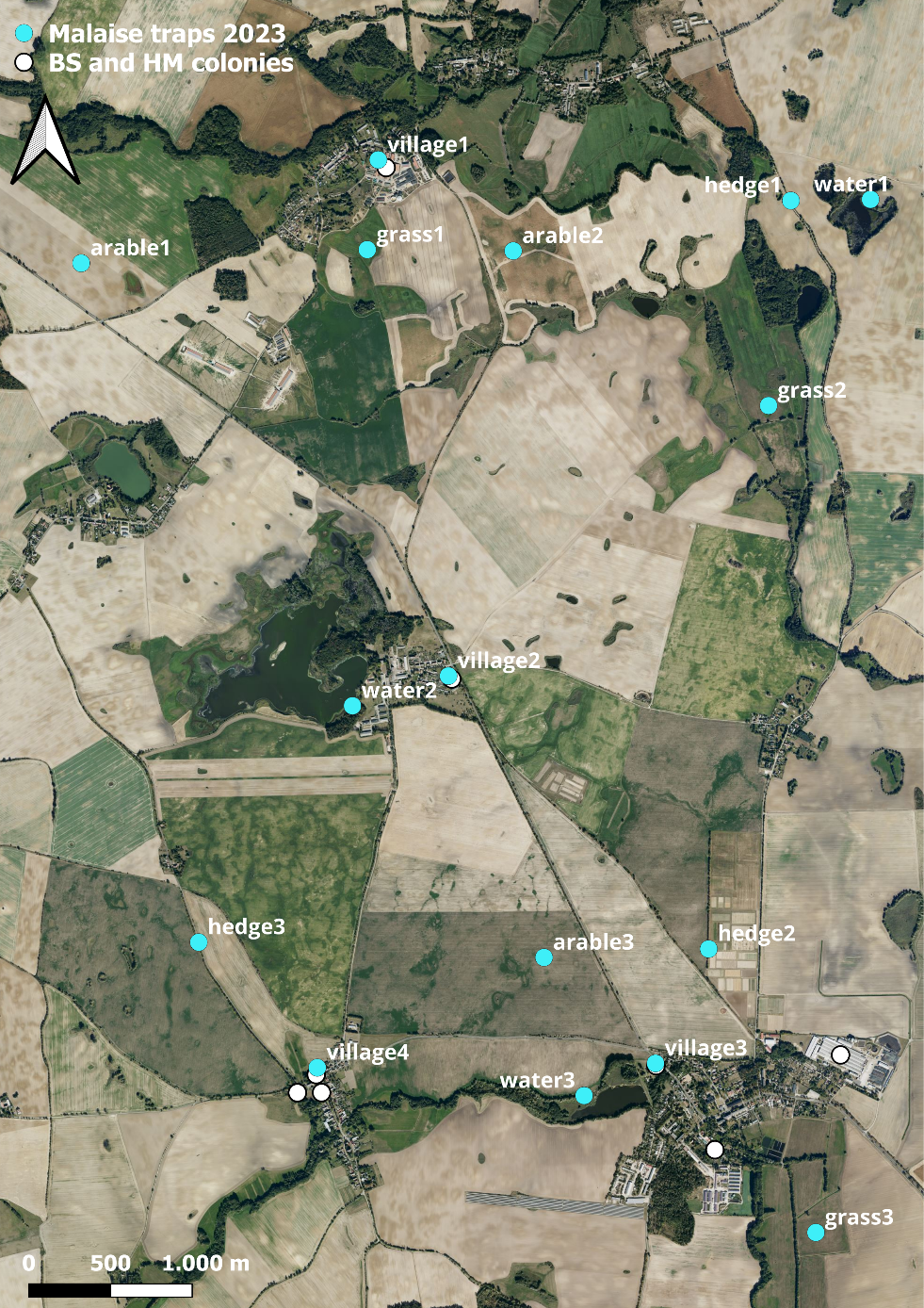


**Fig. S1-5.** Distribution of malaise traps and HM (house martin, *Delichon urbicum*) and BS (barn swallow *Hirundo rustica*) colonies within the study area in 2023. [Google Maps. Map data © Google]

## S1-6. Mapped insects – detailed methods explanation

The information gained from the insect samples (malaise traps) was associated to the corresponding land-use type. For example, for the three malaise traps placed in grasslands, on any given day, we calculated the median biomass (the weight in Gramm of each insect sample) and extrapolated that value for all grasslands in the study area – for that day. We did the same with the median abundance, the median number of taxa (we use the term taxa instead of orders, as we subdivided Diptera into subgroups) and the median insect size for each day and land-use type. Hence, instead of extracting information about the underlying land-use type *per se*, we extracted information about the underlying insect biomass, abundance, taxonomic richness and insect size per land-use type and day. We ran the habitat selection analysis on this daily changing, underlying information. As it was logistically impossible to have malaise traps in all eight land-use types (arable, extensive and intensive grasslands, forest, villages, green areas in villages, water bodies, woody vegetation structures), we used the following combinations to match the malaise trap land-use types to those from the raster: wheat field traps were classified as arable, grassland traps were used for combined grassland (without the distinction between extensive and intensive grasslands), the village traps were used for the combined rural effect (without the distinction between villages and green areas in villages), traps in hedges were used for woody vegetation structures and forests and traps close to water bodies were used for water.

## S1-7. Species-specific distance to colony, including mean and maximum daily displacement information


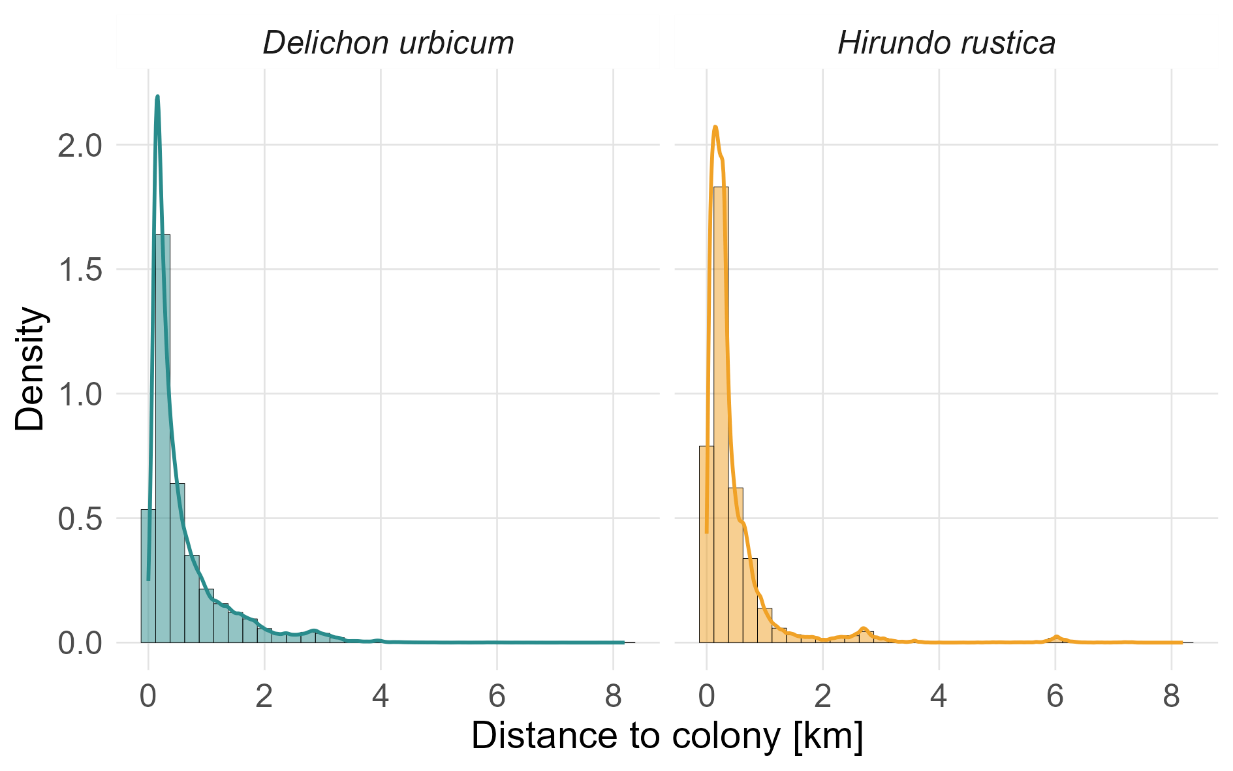


**Fig. S1-7.** Species-specific density histogram for distance to colony (2020-2024), derived from the raw data. Colours represent the species: HM = teal, BS = gold.

**Table S1-7.1.** Fixed effects estimates and 95% confidence intervals (CI) from the Gamma GLMM (log link) testing the differences between mean daily displacement from their colony for the two bird species. The intercept represents the estimated mean daily displacement (in meters) for the reference species (HM) on the response scale. The coefficient for *Hirundo rustica* is shown as a multiplicative ratio relative to HM, such that values < 1 indicate shorter displacement distances in BS. Confidence intervals and p-values are reported on the response scale. Random-effects variance components, sample sizes, and marginal/conditional R² values are shown. Informative parameters are highlighted in bold.

|  | **Mean daily displacement** | | |
| --- | --- | --- | --- |
| *Predictors* | *Estimates* | *CI* | *p* |
| Intercept [HM] | 518.85 | 465.15 – 578.74 | **<0.001** |
| BS | 0.67 | 0.57 – 0.78 | **<0.001** |
| **Random Effects** | | | |
| σ^2^ | 0.19 | | |
| τ_00_ _TAG_ | 0.26 | | |
| N _TAG_ | 197 | | |
| Observations | 929 | | |
| Marginal R^2^ / Conditional R^2^ | 0.083 / 0.618 | | |

**Table S1-7.2.** Fixed effects estimates and 95% confidence intervals (CI) from the Gamma GLMM (log link) testing the differences between maximum daily displacement from their colony for the two bird species. The intercept represents the estimated maximum daily displacement (in meters) for the reference species (HM) on the response scale. The coefficient for *Hirundo rustica* is shown as a multiplicative ratio relative to HM, such that values < 1 indicate shorter displacement distances in BS. Confidence intervals and p-values are reported on the response scale. Random-effects variance components, sample sizes, and marginal/conditional R² values are shown. Informative parameters are highlighted in bold.

|  | **Maximum daily displacement** | | |
| --- | --- | --- | --- |
| *Predictors* | *Estimates* | *CI* | *p* |
| Intercept [HM] | 1810.27 | 1646.53 – 1990.30 | **<0.001** |
| BS | 0.64 | 0.56 – 0.74 | **<0.001** |
| **Random Effects** | | | |
| σ^2^ | 0.24 | | |
| τ_00_ _TAG_ | 0.16 | | |
| N _TAG_ | 197 | | |
| Observations | 929 | | |
| Marginal R^2^ / Conditional R^2^ | 0.109 / 0.470 | | |

## S1-8 Insect abundance per land-use type

| *Land-use type* | *Abundance* |
| --- | --- |
| Arable | 12930 |
| Grasslands | 20869 |
| Hedges | 9538 |
| Villages | 29832 |
| Close to water bodies | 29098 |

## S1-9 Insect abundance per order

| *Order* | *Abundance* |
| --- | --- |
| Brachicera | 57437 |
| Nematocera | 25012 |
| Hymenoptera | 12805 |
| Hemiptera | 3506 |
| Coleoptera | 2155 |
| Lepidoptera | 670 |
| Arachnida | 240 |
| Thysanoptera | 161 |
| Psocodea | 110 |
| Dermaptera | 67 |
| Trichoptera | 42 |
| Neuroptera | 19 |
| Odonata | 14 |
| Orthoptera | 7 |
| Collembola | 6 |
| Ephemeroptera | 6 |
| Raphidioptera | 2 |
|  |  |

## S1-10 Parameter estimates for insect biomass, taxonomic richness and insect body size for the land-use types and distance to nearest habitat edge

**Table S1-10.1:** Fixed effects estimates and 95% confidence intervals (LCI = lower confidence interval, UCI = upper confidence interval) from the GLMMs for insect biomass (log-scale), the taxonomic richness and the average insect size by land-use type (LT) and month (m, June and May). The land-use type arable and the month June are in the intercept. Informative parameters are highlighted in bold.

|  | **log-Abundance** | | | **Taxonomic richness** | | | **Average insect size (mm)** | | |
| --- | --- | --- | --- | --- | --- | --- | --- | --- | --- |
| *Predictors* | *Estimate* | *LCI* | *UCI* | *Estimate* | *LCI* | *UCI* | *Estimate* | *LCI* | *UCI* |
| (Intercept) | **5.05** | **4.48** | **5.62** | **5.49** | **4.95** | **6.03** | **3.36** | **2.95** | **3.78** |
| LT [grassland] | **0.81** | **0.08** | **1.55** | **1.09** | **0.39** | **1.80** | 0.32 | -0.25 | 0.89 |
| LT [village] | **0.74** | **0.05** | **1.43** | **1.48** | **0.82** | **2.14** | **1.02** | **0.49** | **1.56** |
| LT [water] | **1.20** | **0.46** | **1.94** | **2.02** | **1.31** | **2.74** | 0.21 | -0.36 | 0.79 |
| LT [woody] | 0.53 | -0.21 | 1.27 | **2.47** | **1.76** | **3.17** | -0.30 | -0.87 | 0.27 |
| m [May] | 0.15 | -0.31 | 0.61 | **-0.78** | **-1.50** | **-0.06** | 0.13 | -0.28 | 0.54 |
| LT [grassland] × m [May] | -0.08 | -0.54 | 0.38 | -0.43 | -1.36 | 0.50 | 0.25 | -0.31 | 0.80 |
| LT [village] × m [May] | -0.25 | -0.68 | 0.18 | -0.09 | -0.96 | 0.78 | **-0.98** | **-1.50** | **-0.47** |
| LT [water] × m [May] | -0.11 | -0.57 | 0.36 | -0.12 | -1.06 | 0.82 | **-0.63** | **-1.19** | **-0.08** |
| LT [woody] × m [May] | **-1.25** | **-1.71** | **-0.79** | **-1.17** | **-2.10** | **-0.23** | -0.03 | -0.59 | 0.52 |
| **Random Effects** |  |  |  |  |  |  |  |  |  |
| Component | Variance | SD | Column1 | Variance | SD |  | Variance | SD |  |
| Plot | 0.17 | 0.41 |  | 0.02 | 0.15 |  | 0.07 | 0.26 |  |
| Date | 0.11 | 0.32 |  | 0.08 | 0.28 |  | 0.01 | 0.12 |  |
| Residual | 0.32 | 0.57 |  | 1.32 | 1.15 |  | 0.46 | 0.68 |  |
| All three models: | Observations: 251 | | | N_Plot_: 16 |  |  | N_Date_: 16 |  |  |

**Table S1-10.2:** Fixed effects estimates and 95% confidence intervals (LCI = lower confidence interval, UCI = upper confidence interval) from the GLMMs for of insect biomass (log-scale), taxonomic richness and the average insect size depending on distance to the nearest habitat edge (dist. to edge). Informative parameters are highlighted in bold.

|  | **log-Abundance** | | | **Taxonomic richness** | | | **Average insect size (mm)** | | |
| --- | --- | --- | --- | --- | --- | --- | --- | --- | --- |
| *Predictors* | *Estimate* | *LCI* | *UCI* | *Estimate* | *LCI* | *UCI* | *Estimate* | *LCI* | *UCI* |
| (Intercept) | 5.66 | 5.27 | 6.05 | **6.64** | **6.19** | **7.10** | 3.57 | 3,30 | 3,83 |
| dist. to edge | -0.001 | -0.01 | 0.003 | **-0.01** | **-0.01** | **-0.003** | -0.00002 | -0,003 | 0,003 |
| **Random effects** | |  |  |  |  |  |  |  |  |
| Component | Variance | SD |  | Variance | SD |  | Variance | SD |  |
| Plot | 0.36 | 0.60 |  | 0.26 | 0.51 |  | 0.17 | 0,41 |  |
| Date | 0.11 | 0.34 |  | 0.40 | 0.64 |  | 0.02 | 0,14 |  |
| Residual | 0.38 | 0.61 |  | 1.37 | 1.17 |  | 0.53 | 0,73 |  |
| All models: | Observations: 251 | | | N_Plot_: 16 |  |  | N_Date_: 16 |  |  |

## S1-11. logRSS and RSS values for representative and median distances to colony – based on land-use types

**Table S1-11.1:** Estimated *log*-relative selection strength (logRSS), lower and upper 95% confidence intervals (LCI and UCI respectively) and relative selection strength (RSS - exponentiated value of logRSS) of habitat selection by HM and BS for the three different behaviours: commuting, foraging and resting. The information in this table is based on “arable” as the reference land-use category. RSS represents the proportional change in selection relative to arable land (e.g. RSS = 1.3 indicates 30 % higher relative selection). For logRSS the estimated effect is positive (selection) when the values are above zero and negative (avoidance) when the values are below zero. For RSS the selection is above 1 and avoidance below 1. Informative parameters are highlighted in bold. ext. gra = extensively used grassland, int. gra = intensively used grassland, vill.gre = green areas in villages, water = water bodies, woody = woody vegetation structures. Estimates are derived from fixed effects of land-use × species × distance to colony models; all other covariates (distance to water bodies, woody vegetation, and structural edges) were held at their mean, while here we show three representative distances to colony (Dist): 500, 1000 and 2000 m.

|  |  |  | Commuting | | | | Foraging | | | | Resting | | | |
| --- | --- | --- | --- | --- | --- | --- | --- | --- | --- | --- | --- | --- | --- | --- |
| Spe | Land | Dist | logRSS | LCI | UCI | RSS | logRSS | LCI | UCI | RSS | logRSS | LCI | UCI | RSS |
| HM | ext.gra | 500 | **0.179** | **0.037** | **0.321** | **1.196** | **0.103** | **0.028** | **0.178** | **1.109** | **-0.700** | **-1.175** | **-0.225** | **0.497** |
| HM | ext.gra | 1000 | **0.362** | **0.200** | **0.523** | **1.436** | **0.202** | **0.120** | **0.284** | **1.224** | -0.498 | -1.113 | 0.118 | 0.608 |
| HM | ext.gra | 2000 | **0.568** | **0.346** | **0.791** | **1.765** | **0.426** | **0.332** | **0.520** | **1.531** | 0.355 | -0.421 | 1.130 | 1.426 |
| BS | ext.gra | 500 | **0.363** | **0.154** | **0.573** | **1.438** | **0.133** | **0.038** | **0.227** | **1.142** | 0.163 | -0.327 | 0.653 | 1.177 |
| BS | ext.gra | 1000 | **0.422** | **0.131** | **0.712** | **1.524** | 0.019 | -0.122 | 0.159 | 1.019 | 0.229 | -0.505 | 0.963 | 1.257 |
| BS | ext.gra | 2000 | 0.461 | -0.057 | 0.980 | 1.586 | -0.146 | -0.393 | 0.101 | 0.864 | 0.636 | -0.591 | 1.863 | 1.889 |
| HM | forest | 500 | -0.001 | -0.188 | 0.187 | 1.000 | 0.034 | -0.054 | 0.123 | 1.035 | -0.350 | -0.989 | 0.290 | 0.705 |
| HM | forest | 1000 | **0.284** | **0.130** | **0.438** | **1.328** | **0.195** | **0.110** | **0.280** | **1.216** | 0.147 | -0.477 | 0.770 | 1.158 |
| HM | forest | 2000 | **0.648** | **0.424** | **0.872** | **1.912** | **0.492** | **0.394** | **0.589** | **1.635** | 0.671 | -0.098 | 1.440 | 1.957 |
| BS | forest | 500 | 0.238 | -0.096 | 0.573 | 1.269 | **0.273** | **0.160** | **0.386** | **1.313** | **1.114** | **0.339** | **1.888** | **3.045** |
| BS | forest | 1000 | 0.125 | -0.277 | 0.527 | 1.133 | **0.347** | **0.198** | **0.495** | **1.414** | **2.378** | **1.156** | **3.599** | **10.779** |
| BS | forest | 2000 | -0.077 | -0.726 | 0.572 | 0.926 | **0.442** | **0.207** | **0.677** | **1.556** | 1.903 | -0.088 | 3.895 | 6.707 |
| HM | int.gra | 500 | -0.054 | -0.201 | 0.094 | 0.948 | **-0.103** | **-0.179** | **-0.026** | **0.902** | **-1.834** | **-2.351** | **-1.316** | **0.160** |
| HM | int.gra | 1000 | 0.048 | -0.097 | 0.194 | 1.050 | **-0.082** | **-0.163** | **-0.001** | **0.921** | **-2.530** | **-3.229** | **-1.831** | **0.080** |
| HM | int.gra | 2000 | **0.219** | **0.006** | **0.432** | **1.245** | 0.069 | -0.025 | 0.163 | 1.071 | **-2.016** | **-2.862** | **-1.169** | **0.133** |
| BS | int.gra | 500 | **0.233** | **0.046** | **0.419** | **1.262** | **0.164** | **0.071** | **0.258** | **1.179** | -0.305 | -0.863 | 0.252 | 0.737 |
| BS | int.gra | 1000 | 0.127 | -0.109 | 0.363 | 1.135 | **0.259** | **0.153** | **0.366** | **1.296** | 0.265 | -0.516 | 1.047 | 1.304 |
| BS | int.gra | 2000 | -0.049 | -0.465 | 0.366 | 0.952 | **0.409** | **0.257** | **0.562** | **1.506** | -0.009 | -1.415 | 1.397 | 0.991 |
| HM | village | 500 | **0.293** | **0.171** | **0.415** | **1.340** | **0.238** | **0.165** | **0.310** | **1.268** | -0.089 | -0.532 | 0.353 | 0.914 |
| HM | village | 1000 | **0.240** | **0.086** | **0.394** | **1.271** | **0.146** | **0.067** | **0.225** | **1.157** | **-0.952** | **-1.529** | **-0.375** | **0.386** |
| HM | village | 2000 | **0.362** | **0.119** | **0.605** | **1.436** | **0.250** | **0.158** | **0.343** | **1.285** | -0.724 | -1.473 | 0.025 | 0.485 |
| BS | village | 500 | **0.201** | **0.030** | **0.372** | **1.223** | **0.090** | **0.001** | **0.179** | **1.094** | -0.218 | -0.694 | 0.257 | 0.804 |
| BS | village | 1000 | **0.372** | **0.111** | **0.632** | **1.450** | 0.049 | -0.066 | 0.165 | 1.051 | -0.122 | -0.773 | 0.529 | 0.885 |
| BS | village | 2000 | **0.634** | **0.186** | **1.082** | **1.885** | -0.001 | -0.176 | 0.174 | 0.999 | 0.703 | -0.520 | 1.926 | 2.020 |
| HM | vill_gre | 500 | -0.053 | -0.201 | 0.095 | 0.948 | -0.077 | -0.154 | 0.001 | 0.926 | **-1.708** | **-2.236** | **-1.180** | **0.181** |
| HM | vill_gre | 1000 | -0.036 | -0.226 | 0.153 | 0.964 | 0.045 | -0.046 | 0.136 | 1.046 | **-0.957** | **-1.654** | **-0.261** | **0.384** |
| HM | vill_gre | 2000 | 0.031 | -0.292 | 0.354 | 1.032 | **0.284** | **0.180** | **0.388** | **1.328** | 0.465 | -0.438 | 1.368 | 1.592 |
| BS | vill_gre | 500 | -0.186 | -0.403 | 0.032 | 0.830 | **-0.203** | **-0.296** | **-0.111** | **0.816** | **-1.699** | **-2.250** | **-1.147** | **0.183** |
| BS | vill_gre | 1000 | 0.005 | -0.271 | 0.281 | 1.005 | **-0.382** | **-0.508** | **-0.256** | **0.682** | **-2.436** | **-3.258** | **-1.613** | **0.088** |
| BS | vill_gre | 2000 | 0.299 | -0.215 | 0.812 | 1.348 | **-0.615** | **-0.821** | **-0.408** | **0.541** | **-2.169** | **-3.430** | **-0.908** | **0.114** |
| HM | water | 500 | -0.148 | -0.360 | 0.064 | 0.862 | 0.067 | -0.018 | 0.153 | 1.070 | -0.522 | -1.092 | 0.048 | 0.593 |
| HM | water | 1000 | **0.194** | **0.029** | **0.360** | **1.215** | **0.181** | **0.096** | **0.266** | **1.199** | -0.005 | -0.635 | 0.624 | 0.995 |
| HM | water | 2000 | **0.751** | **0.545** | **0.958** | **2.120** | **0.373** | **0.276** | **0.469** | **1.452** | 0.576 | -0.165 | 1.316 | 1.779 |
| BS | water | 500 | 0.317 | -0.025 | 0.659 | 1.373 | **0.200** | **0.089** | **0.310** | **1.221** | -0.070 | -0.694 | 0.554 | 0.932 |
| BS | water | 1000 | **0.563** | **0.291** | **0.834** | **1.756** | **0.376** | **0.255** | **0.497** | **1.457** | 0.666 | -0.133 | 1.465 | 1.946 |
| BS | water | 2000 | **0.901** | **0.469** | **1.332** | **2.461** | **0.610** | **0.398** | **0.821** | **1.840** | **2.363** | **1.257** | **3.469** | **10.622** |
| HM | woody | 500 | -0.083 | -0.241 | 0.074 | 0.920 | -0.010 | -0.086 | 0.067 | 0.990 | **-1.029** | **-1.528** | **-0.529** | **0.357** |
| HM | woody | 1000 | 0.045 | -0.109 | 0.199 | 1.046 | **0.127** | **0.045** | **0.209** | **1.135** | -0.457 | -1.082 | 0.168 | 0.633 |
| HM | woody | 2000 | **0.270** | **0.043** | **0.498** | **1.310** | **0.338** | **0.243** | **0.433** | **1.402** | 0.382 | -0.397 | 1.161 | 1.465 |
| BS | woody | 500 | -0.021 | -0.243 | 0.200 | 0.979 | -0.057 | -0.148 | 0.034 | 0.945 | **-1.145** | **-1.649** | **-0.641** | **0.318** |
| BS | woody | 1000 | -0.055 | -0.312 | 0.203 | 0.947 | -0.095 | -0.205 | 0.016 | 0.910 | **-1.542** | **-2.240** | **-0.844** | **0.214** |
| BS | woody | 2000 | -0.161 | -0.617 | 0.296 | 0.851 | -0.143 | -0.310 | 0.024 | 0.867 | **-1.646** | **-2.754** | **-0.538** | **0.193** |

**Table S1-11.2:** logRSS and RSS values for median distances to colony. Estimated *log*-relative selection strength (logRSS), lower and upper 95% confidence intervals (LCI and UCI respectively) and relative selection strength (RSS - exponentiated value of logRSS) of habitat selection by HM and BS for the three different behaviours: commuting (based on SSF), foraging (based on SSF) and resting (based on RSF). The information in this table is based on “arable” as the reference land-use category. RSS represents the proportional change in selection relative to arable land (e.g. RSS = 1.3 indicates 30 % higher relative selection) for the same species. For logRSS the estimated effect is positive (selection) when the values are above zero and negative (avoidance) when the values are below zero. For RSS the selection is above 1 and avoidance below 1. Informative parameters are highlighted in bold. ext. gra = extensively used grassland, int. gra = intensively used grassland, vill.gre = green areas in villages, water = water bodies, woody = woody vegetation structures. Estimates are derived from fixed effects of land-use × species × distance to colony models; all covariates besides land-use and species (distance to colony, water bodies, woody vegetation, and structural edges) were held at their mean. Mean distance to colony for the three behaviours were: 862 m for commuting, 608 m for foraging and 482 m for resting.

|  | |  | Commuting | | | | | | | Foraging | | | | | | | Resting | | | | | | | |
| --- | --- | --- | --- | --- | --- | --- | --- | --- | --- | --- | --- | --- | --- | --- | --- | --- | --- | --- | --- | --- | --- | --- | --- | --- |
| Spe | Land | | logRSS | | LCI | | UCI | | RSS | logRSS | | LCI | | UCI | | RSS | logRSS | | LCI | | UCI | | RSS | |
| HM | ext.gra | | **0.315** | **0.163** | | **0.468** | | **1.370** | | **0.124** | **0.048** | | **0.200** | | **1.132** | | **-0.700** | **-1.175** | | **-0.225** | | **0.497** | |  |
| BS | ext.gra | | **0.408** | **0.149** | | **0.666** | | **1.503** | | **0.107** | **0.005** | | **0.208** | | **1.113** | | 0.163 | -0.327 | | 0.653 | | 1.177 | |  |
| HM | forest | | **0.210** | **0.060** | | **0.361** | | **1.234** | | 0.070 | -0.016 | | 0.156 | | 1.072 | | -0.350 | -0.989 | | 0.290 | | 0.705 | |  |
| BS | forest | | 0.156 | -0.215 | | 0.528 | | 1.169 | | **0.290** | **0.172** | | **0.408** | | **1.336** | | **1.114** | **0.339** | | **1.888** | | **3.045** | |  |
| HM | int.gra | | 0.021 | -0.119 | | 0.160 | | 1.021 | | -0.101 | -0.178 | | -0.024 | | 0.904 | | **-1.834** | **-2.351** | | **-1.316** | | **0.160** | |  |
| BS | int.gra | | 0.156 | -0.057 | | 0.369 | | 1.168 | | **0.186** | **0.091** | | **0.281** | | **1.204** | | -0.305 | -0.863 | | 0.252 | | 0.737 | |  |
| HM | vill_gre | | -0.042 | -0.213 | | 0.129 | | 0.959 | | -0.050 | -0.130 | | 0.029 | | 0.951 | | **-1.708** | **-2.236** | | **-1.180** | | **0.181** | |  |
| BS | vill_gre | | -0.046 | -0.292 | | 0.200 | | 0.955 | | **-0.245** | **-0.342** | | **-0.148** | | **0.782** | | **-1.699** | **-2.250** | | **-1.147** | | **0.183** | |  |
| HM | village | | **0.248** | **0.106** | | **0.389** | | **1.281** | | **0.210** | **0.137** | | **0.283** | | **1.234** | | -0.089 | -0.532 | | 0.353 | | 0.914 | |  |
| BS | village | | **0.326** | **0.095** | | **0.557** | | **1.385** | | 0.080 | -0.013 | | 0.173 | | 1.084 | | -0.218 | -0.694 | | 0.257 | | 0.804 | |  |
| HM | water | | 0.101 | -0.069 | | 0.272 | | 1.107 | | **0.093** | **0.008** | | **0.177** | | **1.097** | | -0.522 | -1.092 | | 0.048 | | 0.593 | |  |
| BS | water | | **0.498** | **0.223** | | **0.773** | | **1.646** | | **0.241** | **0.134** | | **0.348** | | **1.272** | | -0.070 | -0.694 | | 0.554 | | 0.932 | |  |
| HM | woody | | 0.010 | -0.139 | | 0.158 | | 1.010 | | 0.022 | -0.056 | | 0.099 | | 1.022 | | **-1.029** | **-1.528** | | **-0.529** | | **0.357** | |  |
| BS | woody | | -0.044 | -0.280 | | 0.192 | | 0.957 | | -0.066 | -0.159 | | 0.028 | | 0.936 | | **-1.145** | **-1.649** | | **-0.641** | | **0.318** | |  |

## S1-12. Species-specific vs. landscape dependent sensitivity analyses


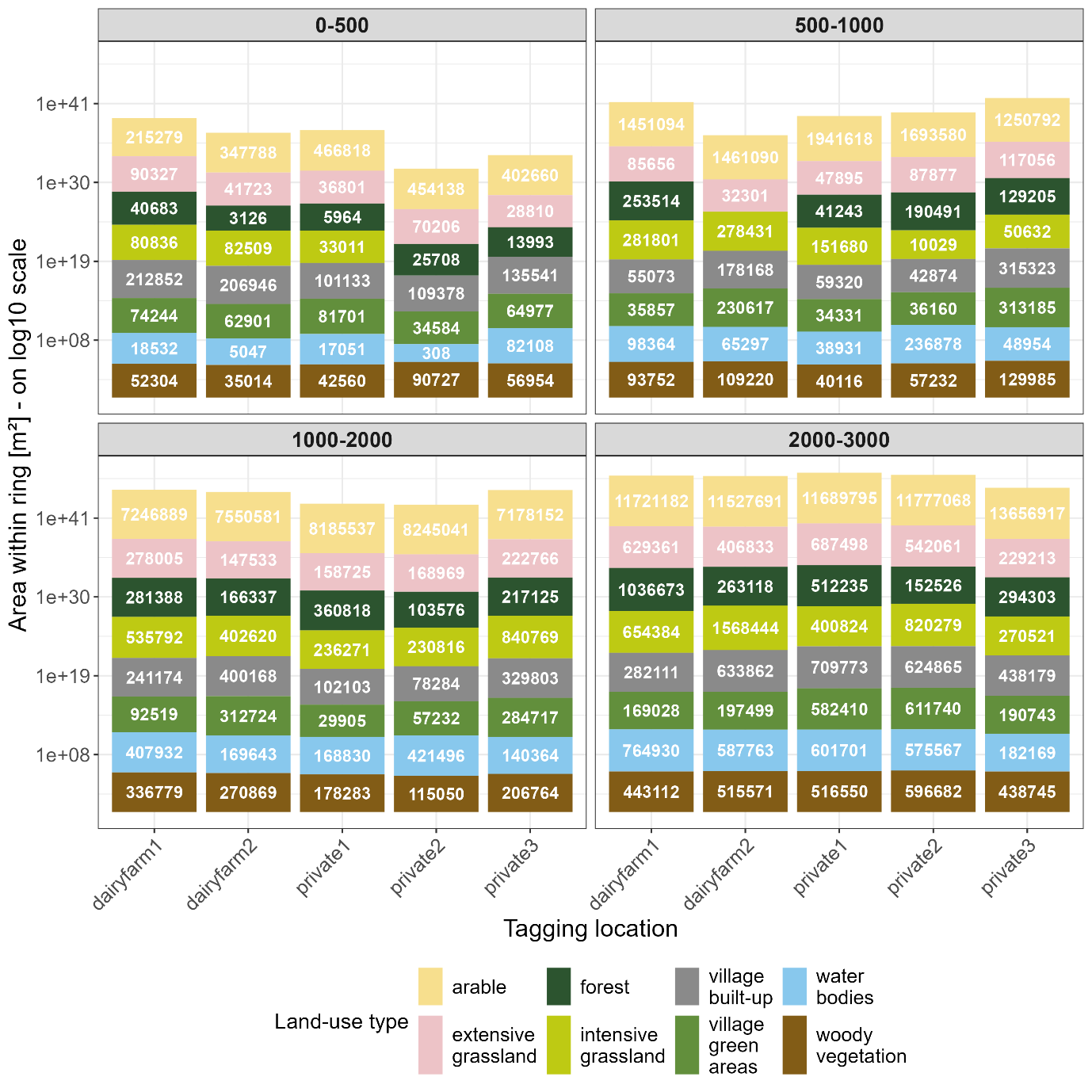


**Fig. S1-12.1.** Absolute area (m², log10‐scaled) of each land‐use type within four concentric distance bands (0–500 m, 500–1000 m, 1000–2000 m, 2000–3000 m) surrounding each tagging location. Bars show the total area of each land‐use type within a ring for five focal tagging locations (three private stables and two dairy farms, the remaining private farms where close to the private farms depicted here and yielded very similar results). Please be aware of the log scale.


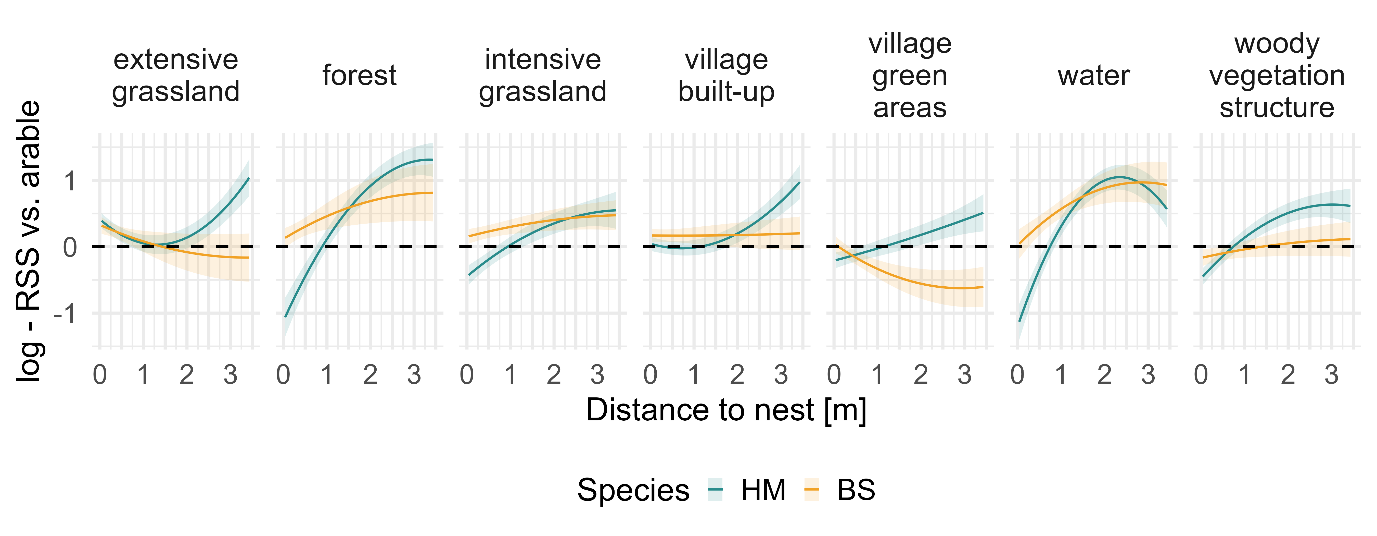


**Fig. S1-12.2.** The effects of the interaction between land-use type and distance from their colony on the logarithmic Relative Selection Strength (log-RSS) for HM (teal) and BS (gold) during foraging for individuals from dairy farm 2 (where both species are present). Values above the dashed horizontal line indicate selection regarding the reference category (arable), while values below the line show avoidance. The ribbons represent the 95% confidence intervals.

## S1-13. logRSS and RSS values for median distances to colony – based on distance to feature


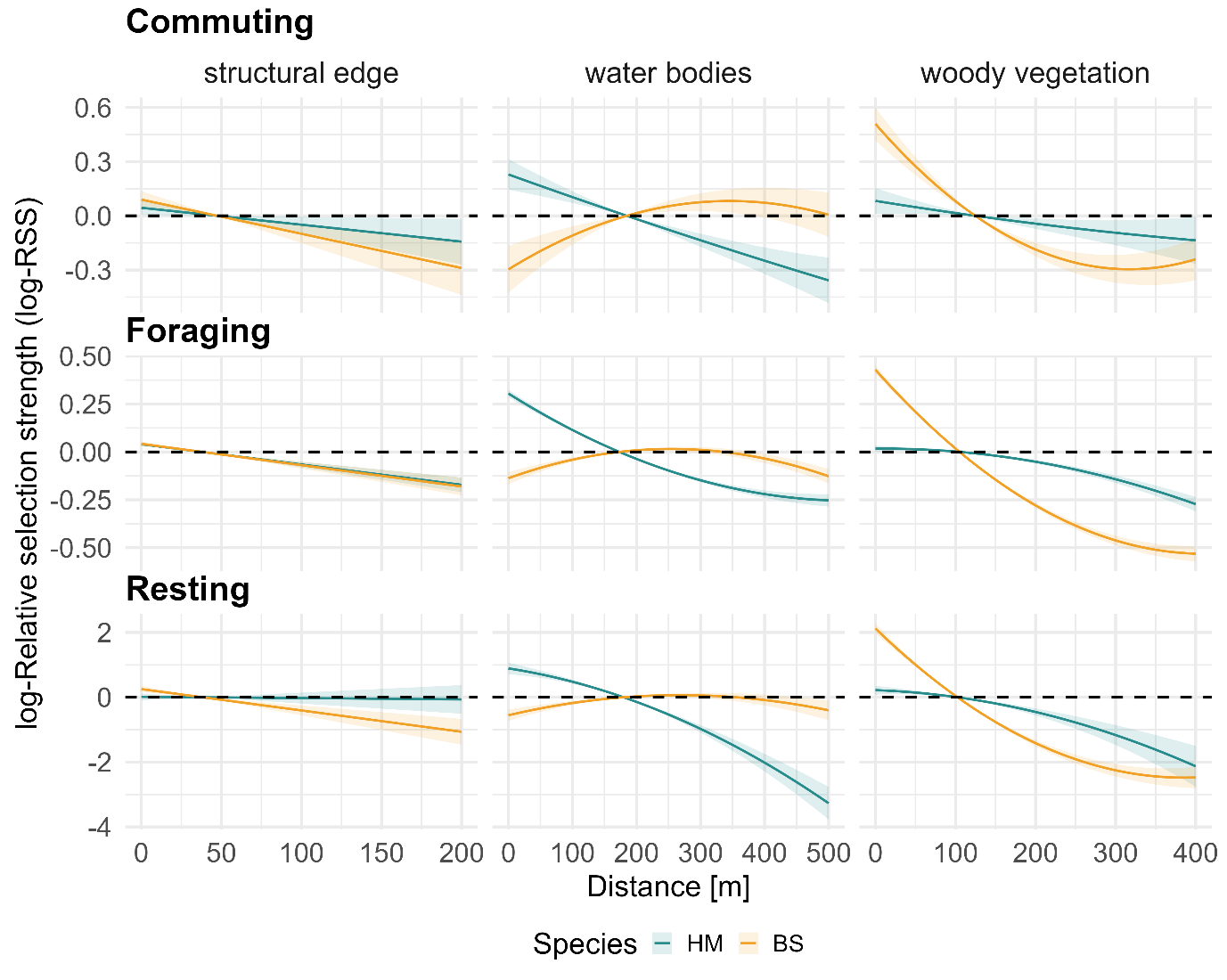


**Fig. S1-13** The effect of distance to structural edges, to water bodies and to woody vegetation structures on the predicted log-Relative Selection Strength for HM (teal) and BS (gold) in the three behaviours: commuting (upper row), foraging (middle row) and resting (lower row). Solid lines show the median predicted log-RSS from the conditional logistic mixed-effects model. Shaded bands denote 95% confidence intervals for fixed effects. All curves are zero‐centred at the average distance to the respective feature, such that values below zero indicate avoidance and above zero indicate preference. The data were obtained from 139 birds for the commuting model, from 172 birds for the foraging model and from 134 birds for the resting model. Note the differing y-axis scales across panels.

**Table S1-13:** logRSS and RSS values for the three distance features at representative distances from the respective feature. Estimated *log*-relative selection strength (logRSS), lower and upper 95% confidence intervals (LCI and UCI respectively) and relative selection strength (RSS - exponentiated value of logRSS) of habitat selection by HM and BS for the three different behaviours: commuting (based on SSF), foraging (based on SSF) and resting (based on RSF). The values are contrasts against a baseline of the same species and behaviour under typical conditions (arable land cover, mean distance to colony, and the distance features that are not the focus in each specific row are also held at their mean). For each distance feature (structural edges (edges), water bodies (water) and woody vegetation (woody)) three representative distances are shown. RSS represents the proportional change in selection relative to arable land (e.g. RSS = 1.3 indicates 30 % higher relative selection). For logRSS the estimated effect is positive (selection) when the values are above zero and negative (avoidance) when the values are below zero. For RSS the selection is above 1 and avoidance below 1. Informative parameters are highlighted in bold.

|  |  |  | Commuting | | | | Foraging | | | | Resting | | | |
| --- | --- | --- | --- | --- | --- | --- | --- | --- | --- | --- | --- | --- | --- | --- |
| Spe. | Feature | Dist | logRSS | LCI | UCI | RSS | logRSS | LCI | UCI | RSS | logRSS | LCI | UCI | RSS |
| HM | Edges | 0 | **0.04** | **0.01** | **0.08** | **1.05** | **0.04** | **0.03** | **0.05** | **1.04** | 0.02 | -0.09 | 0.12 | 1.02 |
| HM | Edges | 50 | 0.00 | -0.01 | 0.00 | 1.00 | **-0.01** | **-0.02** | **-0.01** | **0.99** | 0.00 | -0.04 | 0.03 | 1.00 |
| HM | Edges | 150 | **-0.10** | **-0.18** | **-0.01** | **0.91** | **-0.12** | **-0.14** | **-0.09** | **0.89** | -0.04 | -0.35 | 0.26 | 0.96 |
| BS | Edges | 0 | **0.09** | **0.04** | **0.14** | **1.09** | **0.04** | **0.03** | **0.05** | **1.04** | **0.25** | **0.16** | **0.35** | **1.29** |
| BS | Edges | 50 | -0.01 | -0.01 | 0.00 | 0.99 | **-0.01** | **-0.02** | **-0.01** | **0.99** | **-0.08** | **-0.11** | **-0.05** | **0.92** |
| BS | Edges | 150 | **-0.19** | **-0.29** | **-0.09** | **0.82** | **-0.12** | **-0.16** | **-0.09** | **0.88** | **-0.73** | **-1.01** | **-0.46** | **0.48** |
| HM | Water | 0 | **0.23** | **0.14** | **0.31** | **1.26** | **0.31** | **0.29** | **0.32** | **1.36** | **0.89** | **0.71** | **1.07** | **2.44** |
| HM | Water | 100 | **0.10** | **0.07** | **0.14** | **1.11** | **0.11** | **0.11** | **0.12** | **1.12** | **0.48** | **0.41** | **0.54** | **1.61** |
| HM | Water | 300 | **-0.13** | **-0.17** | **-0.10** | **0.87** | **-0.15** | **-0.16** | **-0.14** | **0.86** | **-0.97** | **-1.08** | **-0.85** | **0.38** |
| HM | Water | 500 | **-0.36** | **-0.48** | **-0.23** | **0.70** | **-0.25** | **-0.29** | **-0.22** | **0.78** | **-3.27** | **-3.78** | **-2.77** | **0.04** |
| BS | Water | 0 | **-0.30** | **-0.43** | **-0.17** | **0.74** | **-0.14** | **-0.17** | **-0.11** | **0.87** | **-0.56** | **-0.73** | **-0.38** | **0.57** |
| BS | Water | 100 | **-0.11** | **-0.16** | **-0.06** | **0.90** | **-0.04** | **-0.05** | **-0.03** | **0.96** | **-0.18** | **-0.24** | **-0.12** | **0.84** |
| BS | Water | 300 | **0.07** | **0.03** | **0.12** | **1.08** | **0.01** | **0.00** | **0.02** | **1.01** | 0.06 | -0.02 | 0.13 | 1.06 |
| BS | Water | 500 | 0.01 | -0.12 | 0.13 | 1.01 | **-0.13** | **-0.16** | **-0.09** | **0.88** | **-0.40** | **-0.71** | **-0.10** | **0.67** |
| HM | Woody | 0 | **0.08** | **0.01** | **0.15** | **1.09** | **0.02** | **0.00** | **0.03** | **1.02** | **0.22** | **0.08** | **0.35** | **1.25** |
| HM | Woody | 100 | **0.02** | **0.00** | **0.03** | **1.02** | **0.00** | **0.00** | **0.00** | **1.00** | **0.01** | **0.00** | **0.01** | **1.01** |
| HM | Woody | 300 | **-0.09** | **-0.16** | **-0.03** | **0.91** | **-0.14** | **-0.16** | **-0.12** | **0.87** | **-1.16** | **-1.47** | **-0.86** | **0.31** |
| HM | Woody | 400 | **-0.14** | **-0.27** | **-0.01** | **0.87** | **-0.27** | **-0.31** | **-0.23** | **0.76** | **-2.13** | **-2.76** | **-1.50** | **0.12** |
| BS | Woody | 0 | **0.51** | **0.42** | **0.60** | **1.66** | **0.43** | **0.41** | **0.45** | **1.54** | **2.12** | **1.98** | **2.27** | **8.37** |
| BS | Woody | 100 | **0.08** | **0.06** | **0.09** | **1.08** | **0.02** | **0.02** | **0.02** | **1.02** | **0.04** | **0.03** | **0.04** | **1.04** |
| BS | Woody | 300 | **-0.29** | **-0.37** | **-0.22** | **0.75** | **-0.46** | **-0.49** | **-0.44** | **0.63** | **-2.25** | **-2.44** | **-2.06** | **0.11** |
| BS | Woody | 400 | **-0.24** | **-0.36** | **-0.12** | **0.79** | **-0.53** | **-0.57** | **-0.49** | **0.59** | **-2.47** | **-2.80** | **-2.14** | **0.08** |

## S1-14 AIC table comparing the mapped insect habitat selection metrics and corresponding PCA bioplot

**Table S1-14:** The performance metrics (logLik, AIC, BIC, deviance and delta AIC) of the habitat selection models for the land-use types, the three insect metrics and the two principal component axes derived from a metrics-based PCA. These habitat selection models were based on the 2023 data set only.

| *model* | *df* | *logLik* | *AIC* | *BIC* | *deviance* | ∆*AIC* |
| --- | --- | --- | --- | --- | --- | --- |
| land-use type | 58 | -1076924 | 2153965 | 2154629 | 290206 | 0 |
| taxonomic richness | 22 | -1077471 | 2154986 | 2155238 | 291299 | 1021 |
| PC2 | 22 | -1077567 | 2155177 | 2155429 | 291490 | 1212 |
| PC1 | 22 | -1077569 | 2155182 | 2155433 | 291495 | 1217 |
| size | 22 | -1077605 | 2155253 | 2155505 | 291566 | 1288 |
| abundance | 22 | -1077607 | 2155258 | 2155509 | 291571 | 1293 |


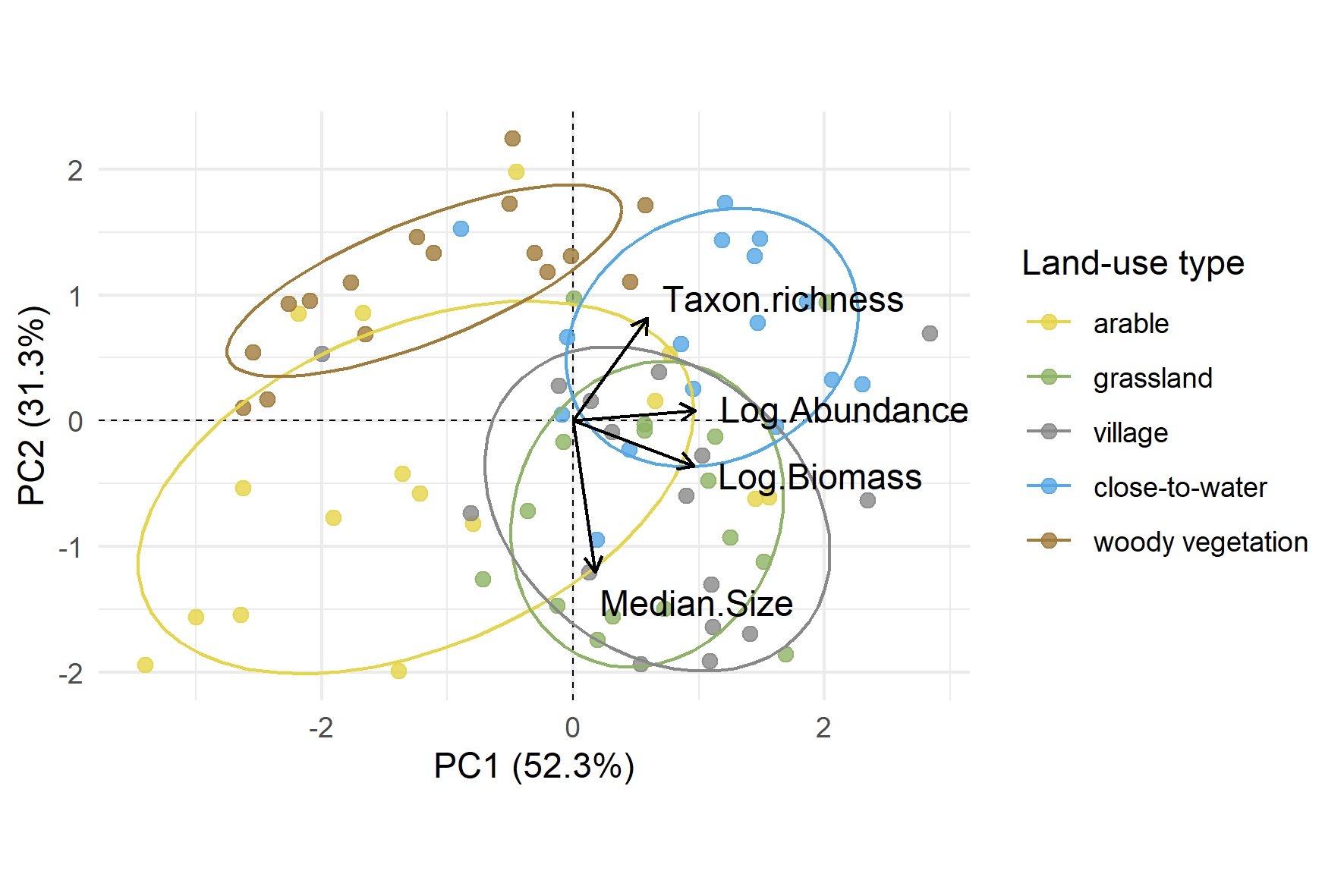


**Fig. S1-14.1.** Principal component analysis (PCA) of daily insect community metrics across five land-use types. Points represent site-day combinations, coloured by land-use category (arable*,* grassland*,* village*,* close-to-water*,* woody vegetation); ellipses show 68% confidence regions. Axes correspond to PC1 (52.3% variance explained) and PC2 (31.3%). Vectors indicate loadings for log-transformed biomass, log-transformed abundance, taxon richness, and median insect body size.


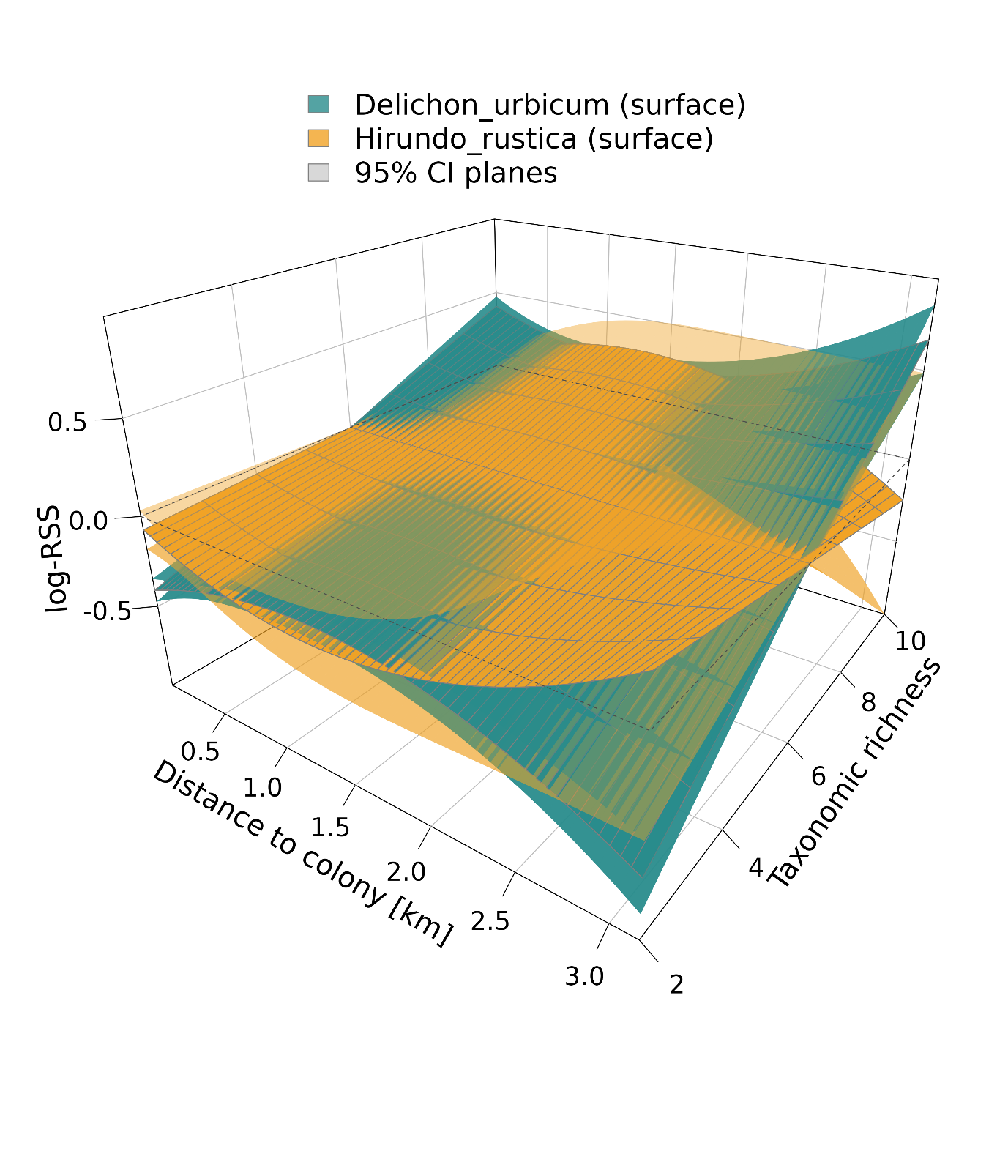


**Fig. S1-14.2.** The effects of the interaction between hirundine species, the number of insect taxa and distance from the colony on the logarithmic Relative Selection Strength (log-RSS) for HM (teal) and BS (gold). Values above the dashed horizontal line indicate selection, while values below the line show avoidance.

## Sensitivity analysis for the buffer size around resting locations


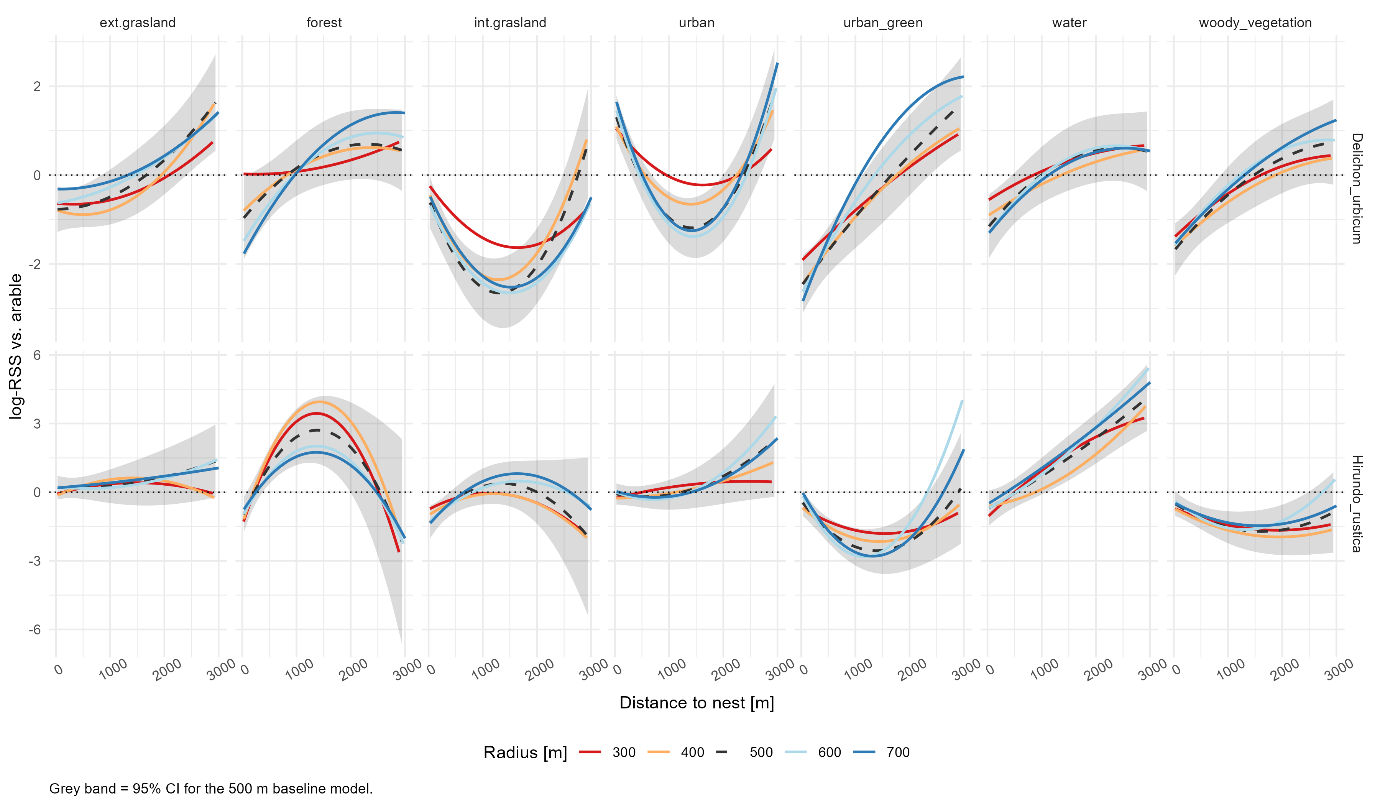


**Fig. S1-15.** The effects of the interaction between land-use type and distance from their colony on the logarithmic Relative Selection Strength (log-RSS) for HM (Delichon_urbicum, upper row) and BS (Hirundo_rustica, lower row) during resting, for different radii around the last movement location (300 m, 400 m, 500 m, 600 m, 700 m). The grey ribbons represent the 95% confidence intervals of the 500 m baseline model. (urban = village)
